# Risk of developing depression from endocrine treatment: A nationwide cohort study of women administered treatment for breast cancer in South Korea

**DOI:** 10.3389/fonc.2022.980197

**Published:** 2022-09-20

**Authors:** Jooyoung Oh, Hye Sun Lee, Soyoung Jeon, Dooreh Kim, Jeong-Ho Seok, Woo-Chan Park, Jae-Jin Kim, Chang Ik Yoon

**Affiliations:** ^1^ Department of Psychiatry, Gangnam Severance Hospital, Yonsei University College of Medicine, Seoul, South Korea; ^2^ Institute of Behavioral Sciences in Medicine, Yonsei University College of Medicine, Seoul, South Korea; ^3^ Biostatistics Collaboration Unit, Yonsei University College of Medicine, Seoul, South Korea; ^4^ Division of Breast Surgery, Department of Surgery, Seoul St Mary’s Hospital, College of Medicine, The Catholic University of Korea, Seoul, South Korea

**Keywords:** breast cancer, endocrine treatment, tamoxifen, aromatase inhibitor, depression

## Abstract

**Background:**

Although previous studies demonstrated no association between depression and tamoxifen in patients with breast cancer, there is still a limited amount of long-term follow-up data. This study aimed to evaluate the relationship between endocrine treatment and the risk of depression.

**Methods:**

This nationwide population-based cohort study used data obtained over a 14-year period (January 2007 to December 2021) from the Korean National Health Insurance claims database. All female patients with breast cancer were included. We examined the incidence of depression in patients who underwent endocrine treatment, and those who did not undergo endocrine treatment constituted the control group.

**Results:**

The data from 11,109 patients who underwent endocrine treatment and 6,615 control patients between 2009 and 2010 were analyzed. After performing matching for comorbidities and age, both groups comprised 6,532 patients. The median follow-up were 119.71 months. Before and after matching was performed, the endocrine treatment was not a significant risk factor for developing depression (*p=*0.7295 and *p=*0.2668, respectively), nor was it a significant factor for an increased risk for suicide attempt (*p*=0.6381 and *p*=0.8366, respectively).

**Conclusions:**

Using a real-world population-based cohort, this study demonstrated that there is no evidence that the endocrine treatment increases the risk of depression.

## Introduction

Depression is more common in cancer patients than in the general population. The prevalence of major depressive disorder is 3.3% in the general population, whereas it is approximately four times higher (12.5%) in cancer patients (1). The prevalence of depression and other diagnoses of depression, including dysthymia and minor depression, is 20% in patients with malignancy (2, 3). Depression is a significant independent risk factor for overall survival regardless of the tumor-node-metastasis stage and affected organs (4).

Breast cancer is the most common type in women, with more than one million cases reported annually worldwide. From the diagnosis of breast cancer to long-lasting and uncertain treatment, breast cancer causes great stress to patients, leading to psychological instability. Anti-estrogen agents (tamoxifen and aromatase inhibitor [AI]) are used to prevent the recurrence of hormone receptor-positive breast cancer (5, 6) however, endocrine treatment may increase the risk of depression symptoms (6). A previous large cohort study demonstrated no increased rate of depression in patients treated with tamoxifen compared with other therapies, despite earlier opposing reports. The National Surgical Adjuvant Breast and Bowel Project (NSABP) P-1 trial used a questionnaire on depression and reported that there was no difference in the degree of depression according to tamoxifen administration during the 3-year follow-up period (7). Nevertheless, several articles have reported that endocrine treatment in patients with breast cancer has a high prevalence of mood disturbances, including depression (8, 9). In addition, there is minimal long-term follow-up data on depression from endocrine treatment.

Therefore, we aimed to identify the risk of depression in patients with breast cancer from endocrine treatment using a nationwide population-based cohort registry. Furthermore, we evaluated the difference in the incidence of depression according to the diagnosis type (invasive breast cancer or ductal carcinoma *in situ* [DCIS]), and endocrine regimen administered.

## Methods

### Study design and database

This retrospective observational cohort study used data from the Korean National Health Insurance (NHI) claims database of the Health Insurance Review and Assessment Service (HIRA), the public institution responsible for evaluating the medical expenses of all medical institutions, in South Korea over a 15-year period from January 2007 to December 2021.The NHI database includes all medical data, such as personal information, date of disease registry, diagnostic codes, procedure information, and prescription information. The disease codes specified in the International Classification of Disease, 10^th^ revision (ICD-10) (10) were used to record the diagnoses.

In this study, all individual information was anonymized prior to performing the data processing to comply with the privacy guidelines of the Health Insurance Portability and Accountability Act. The study protocol was approved by the institutional review board (IRB) of Gangnam Severance Hospital (local IRB no.: 3-2021-0050), which waived the need for informed consent based on the retrospective cohort design.

### Study population

All women aged ≥20 years in the NHI database were included in the initial screening stage. We included patients diagnosed with DCIS (ICD-10 code, D05) or breast cancer (ICD-10 code, C50) between 2009 and 2010 and who had not seen a physician for any type of cancer (ICD-10 code; any C code) during the 2-year washout period between 2007 and 2008. To minimize misclassification errors, patients with breast cancer or DCIS were defined only as those with a behavior surgical code within 1 year of receiving the first diagnosis code (**Supplementary Table 1**). The day of enrollment for all subjects was the day of the surgery for breast cancer. The follow-up months were calculated from the date of the enrollment date. The participants were monitored for the occurrence of depression until 2021. Patients without an event were censored on December 31, 2021. We evaluated the risk of developing depression from endocrine therapy in breast cancer patients.

### Predictor and outcome variables

The primary outcome of interest was the occurrence of depression (ICD-10 codes F32 and F33) from endocrine treatment between January 1, 2011, and December 31, 2021. These F32 and F33 codes included mild to severe single depressive episodes with or without psychotic symptoms, mild to severe recurrent depressive episodes with or without psychotic symptoms, and other minor types of depression; therefore, these two codes cover the overall depression diagnoses.

The secondary outcome was at least one suicide attempt or probable suicide attempt accompanied by depression from endocrine treatment during the observation period of January 1, 2011, to December 31, 2021. Individuals who experienced self-harm were categorized into two groups. Suicide attempters were defined by the following diagnostic codes (ICD-10 codes: intentional self-harm, X60-X85). The probable suicide attempters included the following ICD-10 codes: wrist laceration (S61.9); poisoning by psychotropic drugs (T43); acute drug intoxication (T50.9); toxic effects of organic solvents, corrosive substances, carbon monoxide, pesticides, other specified substances, and unspecified substances (T52, T54, T58, T60, and T65); handgun, rifle, or other firearm discharges (W32, W33, and W34); accidental suffocation and strangulation in bed (W75); other accidental hanging and strangulation (W76); event of undermined intent (Y10-Y34), asphyxia (R09); and falls (W13-W19) (**Supplementary Table 2**). The first event for individuals who committed multiple suicide attempts or probable suicide attempts was analyzed in our study.

Subgroup analyses were performed, including only patients who had been diagnosed with depression and were taking antidepressants [depression diagnosis with an antidepressant prescription (**Supplementary Table 3**)]. We also conducted a subgroup analysis according to the severity of the cancer diagnosis (invasive breast cancer versus DCIS) and that according to the endocrine regimen used (tamoxifen versus AI) (**Supplementary Table 4**).

To minimize misclassification errors, the patients diagnosed with depression with the following diseases were excluded from the analysis: mental retardation (ICD-10 code: F7), schizophrenia (F2), bipolar disorder (F30 and F31), and epilepsy (G40 and G41).

### Confounding variables

The confounding variables included several comorbidities, medications, receipt of chemotherapy (**Supplementary Table 5**), and age. The confounding variables in this study were defined in relation to the development of depression, as follows: goiter (ICD-10 code: E01), hypothyroidism (E02-E03), thyrotoxicosis (E05), thyroiditis (E06), endocrine disorder (E89), medications (levothyroxine or steroid, **Supplementary Table 6**), diabetes (E10-E14), hypertension (I10), hyperlipidemia (E78), chronic obstructive pulmonary disease (COPD; J44), chronic kidney disease (CKD; N18), liver cirrhosis (K74 and K703), and heart failure (I50). Furthermore, we defined the presence of comorbidities as any diagnosis of the aforementioned codes in the 2 years preceding the enrollment date.

To minimize selection bias, estimated propensity scores were used to match the patients with breast cancer who received endocrine therapy to those who did not. This was calculated for each participant using logistic regression analysis with the variables of age, medications, and comorbidities. A nearest neighbor greedy algorithm was used to match the subjects using the propensity scores. We also used 1:1 propensity score-matching to obtain the maximum number of patients with breast cancer and minimize the estimated results (11). We tested the proportional-hazards assumption by including an interaction term between variables and natural-log–transformed follow-up time. We also checked by log-minus-log survival plots. We investigated the association between the endocrine medication (tamoxifen or AI) and the incidence of depression in patients with breast cancer. In addition, subgroup analyses were performed to identify the differences in the risk of developing depression between those with invasive breast cancer and those with DCIS.

### Statistical analysis

To compare the characteristics between the two groups, Student’s *t*-test was used to analyze continuous variables, and the chi-square test or Fisher’s exact test was used to analyze categorical variables. The cumulative depression incidence rates for the two groups were obtained using Kaplan–Meier curves and compared using the log-rank test. Cox proportional hazard models were used to determine the hazard ratio (HR) and 95% confidence interval (CI) to investigate the onset of depression after adjusting for the confounding variables. We applied the backward likelihood method (entering effects: *p*=0.05; removing effects: *p*=0.05). Statistical significance was set at a two-sided *p-*value of <0.05. Statistical analyses were performed using SAS software (version 9.4, SAS Institute, Cary, NC, USA).

## Results

### Characteristics of the registry

**Figure 1:**
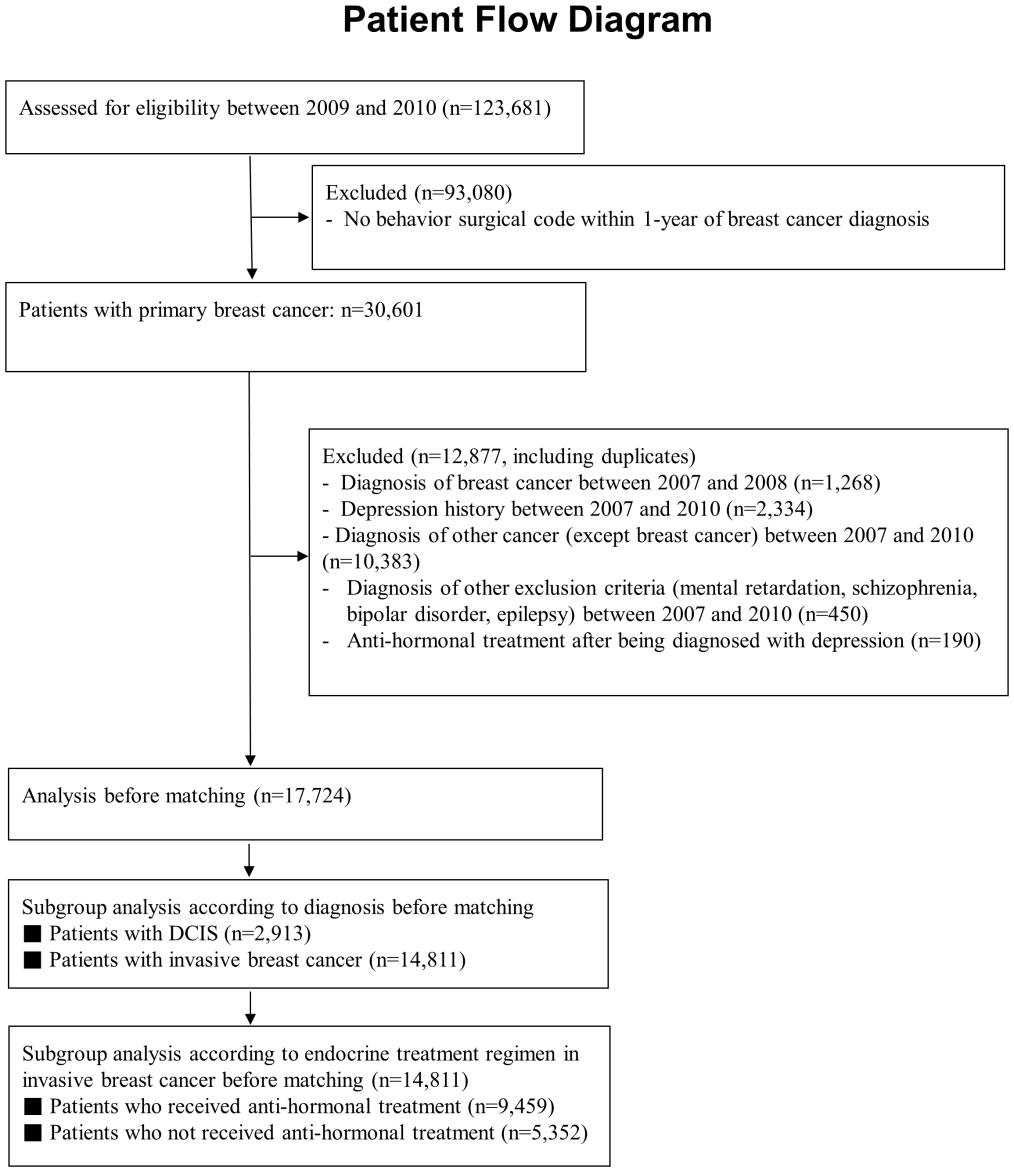
Flow diagram for the selection and enrollment of eligible patients in this study.

In total, 123,681 patients with breast cancer were included in this study (**Figure 1**). We excluded patients without a surgical behavior code (to select only new primary breast cancer patients without metastasis) within 1 year of the breast cancer diagnosis. After excluding patients who were diagnosed with other malignancies, depression or other disorders (mental retardation etc.) before 2010, 17,724 patients with breast cancer were included in study sample. After matching, the endocrine treatment and control groups comprised 6,532 patients (**Table 1**). Of the total patients, 2,847 (16.1%) were diagnosed with depression, and 2,301 (13.0%) were prescribed antidepressants. Among the patients, 274 (1.6%) attempted suicide or had a probable suicide attempt, of whom 37 (0.2%) were diagnosed with depression at the same time. **Table 1** shows the between-group differences in comorbidities, levothyroxine/steroid use, chemotherapy and age. After performing matching, both groups were well-balanced in comorbidities, drug history, chemotherapy use, and age. During the matching process, all variables met proportional-hazards assumption.

**Table 1:** Comparison of clinical characteristics of patients with breast cancer and DCIS according to receipt of endocrine treatment.

	Before matching			After matching		
	Patients not receiving endocrine treatment, n = 6,615 (%)	Patients receiving endocrine treatment, n = 11,109 (%)	*P* value	Patients not receiving endocrine treatment, n = 6,532 (%)	Patients receiving endocrine treatment, n = 6,532 (%)	*P* value
**Depression (only diagnosis)**			0.629			0.706
** No**	5,541 (83.8)	9,336 (84.0)		5,468 (83.7)	5,452 (83.5)	
** Yes**	1,074 (16.2)	1,773 (16.0)		1,064 (16.3)	1,080 (16.5)	
**Depression (diagnosis + anti-depressant)**			0.810			0.642
** No**	5,751 (86.9)	9,672 (87.1)		5,676 (86.9)	5,658 (86.6)	
** Yes**	864 (13.1)	1,437 (12.9)		856 (13.1)	874 (13.4)	
**Depression (only diagnosis) + suicidal attempt**			0.456			>.999
** No**	6,599 (99.8)	11,088 (99.8)		6,516 (99.8)	6,516 (99.8)	
** Yes**	16 (0.2)	21 (0.2)		16 (0.24)	16 (0.2)	
**Depression (diagnosis + anti-depressant) + suicidal attempt**						0.705
** No**	6,600 (99.8)	11,091 (99.8)	0.334	6,517 (99.8)	6,519 (99.8)	
** Yes**	15 (0.2)	18 (0.2)		15 (0.2)	13 (0.2)	
**Suicidal attempt**			0.638			0.837
** No**	6,509 (98.4)	10,941 (98.5)		6,426 (98.4)	6,423 (98.3)	
** Yes**	106 (1.6)	168 (1.5)		106 (1.62)	109 (1.7)	
**Endocrine disorder (including thyroid disease)**			0.153			0.759
** No**	6,028 (91.1)	10,192 (91.8)		5,956 (91.2)	5,946 (91.0)	
** Yes**	587 (8.9)	917 (8.3)		576 (8.8)	586 (9.0)	
**Taking synthroid**			0.936			0.545
** No**	6,415 (97.0)	10,783 (97.1)		6,335 (97.0)	6,323 (96.8)	
** Yes**	200 (3.0)	326 (2.9)		197 (3.0)	209 (3.2)	
**Taking steroids**			0.314			0.708
** No**	2,135 (32.3)	3,667 (33.0)		2,114 (32.4)	2,094 (32.1)	
** Yes**	4,480 (67.7)	7,442 (67.0)		4,418 (67.6)	4,438 (67.9)	
**Diabetes**			0.057			0.454
** No**	5,834 (88.2)	9,901 (89.1)		5,785 (88.6)	5,812 (89.0)	
** Yes**	781 (11.8)	1,208 (10.9)		747 (11.4)	720 (11.0)	
**Hypertension**			0.559			0.739
** No**	5,100 (77.1)	8,607 (77.5)		5,044 (77.2)	5,028 (77.0)	
** Yes**	1,515 (22.9)	2,502 (22.5)		1,488 (22.8)	1,504 (23.0)	
**Hyperlipidemia**			0.061			0.385
** No**	5,400 (81.6)	9,192 (82.7)		5,352 (81.9)	5,390 (82.5)	
** Yes**	1,215 (18.4)	1,917 (17.3)		1,180 (18.1)	1,142 (17.5)	
**COPD**			0.842			0.318
** No**	6,385 (96.5)	10,729 (96.6)		6,313 (96.7)	6,292 (96.3)	
** Yes**	230 (3.5)	380 (3.4)		219 (3.4)	240 (3.7)	
**CKD**			0.020			0.114
** No**	6,576 (99.4)	11,070 (99.7)		6,497 (99.5)	6,509 (99.7)	
** Yes**	39 (0.6)	39 (0.4)		35 (0.5)	23 (0.4)	
**LC**			0.698			0.752
** No**	6,590 (99.6)	11,071 (99.7)		6,511 (99.7)	6,513 (99.7)	
** Yes**	25 (0.4)	38 (0.3)		21 (0.3)	19 (0.3)	
**Heart failure**			0.029			0.621
** No**	6,557 (99.1)	11,043 (99.4)		6,483 (99.3)	6,478 (99.2)	
** Yes**	58 (0.9)	66 (0.6)		49 (0.8)	54 (0.8)	
**Chemotherapy**			0.181			0.778
** Not done**	2,912 (44.0)	4,776 (43.0)		2,860 (43.8)	2,844 (43.5)	
** Done**	3,703 (56.0)	6,333 (57.0)		3,672 (56.2)	3,688 (56.5)	
**Age (year, mean ± SD)**	50.83 ± 11.29	50.34 ± 10.32	0.004	50.70 ± 11.22	50.77 ± 10.77	0.735

COPD, chronic obstruction pulmonary disease; CKD, chronic kidney disease; LC, liver cirrhosis; SD, standard deviation.

### Analysis of risk of developing depression

**Figure 2:**
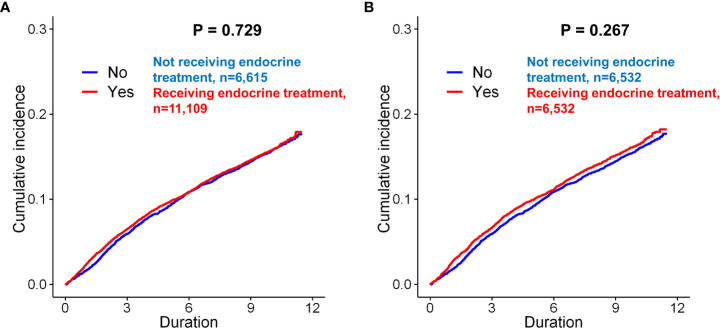
Kaplan–Meier analysis of the incidence of depression in breast cancer patients according to endocrine treatment. Before matching, there was no significant difference between the patients who underwent endocrine treatment and those who did not undergo endocrine treatment **(A**, *p* = 0.729, log-rank test**)**. After matching, there was still no significant difference between the two groups of patients **(B**, *p* = 0.267**)**.

Before matching, the mean and median follow-up times for the total patients were 121.91 ± 7.77 and 121.57 (97.2–137.5) months, respectively. Before matching, depression (only diagnosis) occurred in 1,074 (16.2%) of 6,615 and 1,773 (16.0%) of 11,109 patients in the control and endocrine treatment groups, respectively (*p*=0.629, **Table 1**). There was no significant difference in the incidence rates between patients received endocrine treatment and those not received endocrine therapy (*p=*0.729, **Figure 2A**). After matching, depression was exhibited in 1,064 (16.3%) of 6,532 and 1,080 (16.5%) of 6,532 patients in the control and endocrine treatment groups, respectively (*p*=0.706, **Table 1**). There was no significant difference in the prevalence of depression between patients who received endocrine treatment and those who did not (*p=*0.267, **Figure 2B**).

In the univariate Cox proportional hazard analyses of the unmatched cohorts, endocrine treatment was not associated with depression (*p*=0.730). In matched cohorts, previous endocrine treatment (*p=*0.267) was not a significant predictor for depression after adjusting for the other variables.

Before matching, depression events (diagnosis+anti-depressant) occurred in 864 (13.1%) of the 6,615 controls and in 1,437 (12.9%) of the 11,109 patients who underwent endocrine treatment (*p*=0.810, **Table 1**). After matching, the patients who underwent endocrine treatment did not show a statistically significant difference in the incidence of depression compared to the controls (*p*=0.642). In Kaplan-Meier curves, there was no significant difference in the prevalence of depression between patients who received endocrine treatment and those who did not (**Supplementary Fig 1**). In the univariate analysis before and after matching, endocrine treatment was not a significant risk factor for depression (before matching, *p*=0.590; after matching, *p*=0.249).

### Sensitivity analysis of risk of depression according to endocrine regimens

**Figure 3:**
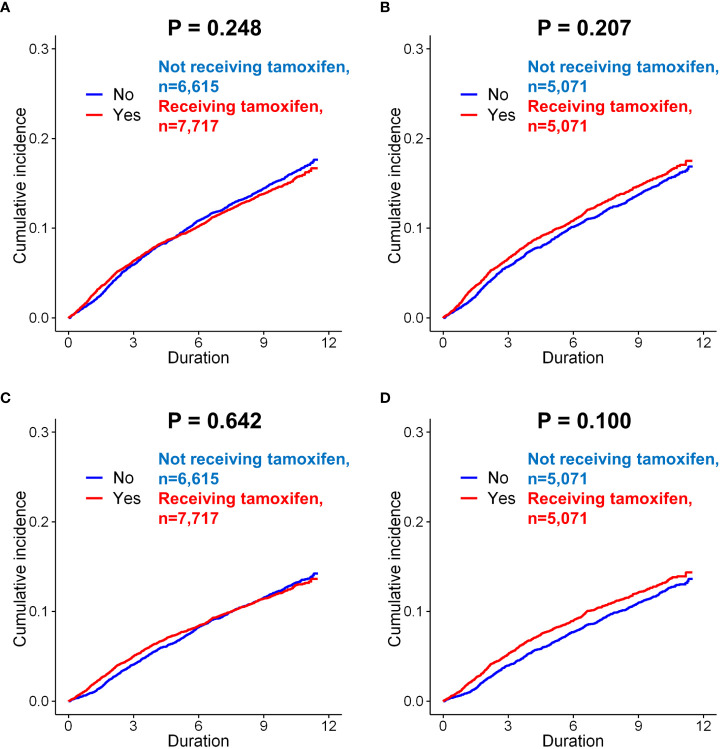
Subgroup analysis of incidence of depression in breast cancer patients according to the use of tamoxifen. Before matching, there was no difference in the incidence (only diagnosis) between the patients administered tamoxifen and those not administered tamoxifen **(A**, *p* = 0.248, log-rank test**)**. After matching, there was still no significant difference between these two groups **(B**, *p* = 0.207**)**. When the incidence of depression (diagnosis + anto-depressant) analyzed in patients, there was no difference in the risk of depression according to tamoxifen use, both before matching **(C**, *p* = 0.642**)** and after matching **(D**, *p* = 0.100**)**.

The effect of endocrine treatment on the incidence of depression was evaluated in patients with breast cancer and in those with DCIS (**Supplementary Fig 6**). In tamoxifen use group, incidence of depression (only diagnosis) were in 1,074 (16.2%) of 6,615 and 1,165 (15.1%) of 7,717 patients in the control and tamoxifen use groups, respectively (*p*=0.061, **Supplementary Table 7**), before matching. There was no significant difference for depression between two groups (*p=*0.248, **Figure 3A**). There was no statistically significant difference for depression between two groups after matching (*p=*0.207, **Figure 3B**). Tamoxifen use was not associated with the risk of depression (before matching, *p=*0.247; after matching, *p=*0.207). The incidence of depression (diagnosis + antidepressants) was also analyzed in patients who underwent tamoxifen therapy. Tamoxifen use was not significantly associated with the risk of depression (before matching, *p*=0.642, **Figure 3C**; after matching, *p*=0.100, **Figure 3D**). In the Cox proportional hazard model, tamoxifen use was not a significant determinant of depression (diagnosis + antidepressants) before or after matching.

**Figure 4:**
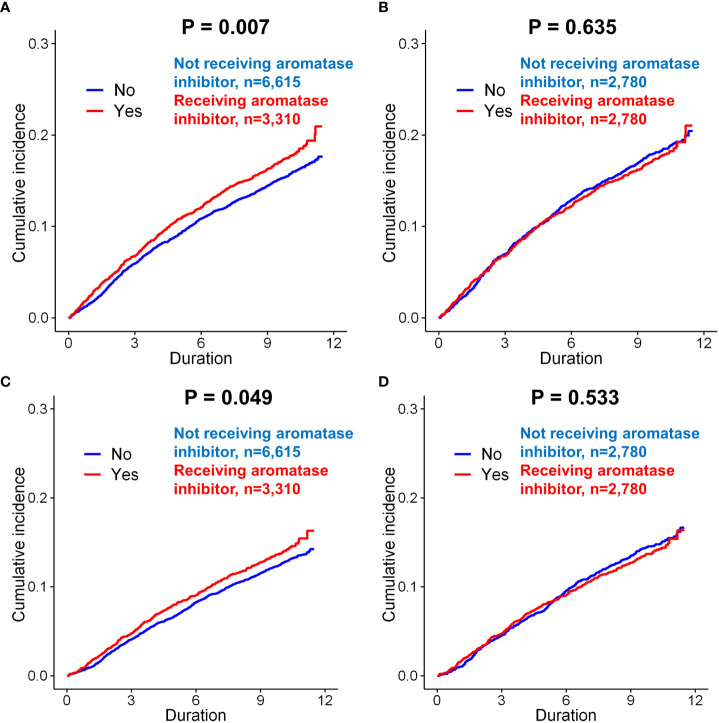
Subgroup analysis of incidence of depression in breast cancer patients according to the use of aromatase inhibitors. Before matching, patients administered aromatase inhibitors was exhibited a higher incidence of depression (only diagnosis) than those not administered aromatase inhibitors **(A**, *p* = 0.007**)**. However, there was no significant difference between these two groups after matching **(B**, *p* = 0.635**)**. When the incidence of depression (diagnosis + anto-depressant) was analyzed in the patients, the patients administered aromatase inhibitors had a signficantly increased risk of depression than those not administered aromatase inhibitors **(C**, *p* = 0.049**)**. However, there was no significant difference between these two groups after matching **(D**, *p* = 0.533**)**.

The effect of AI use on the incidence of depression was evaluated in patients with invasive breast cancer. Before matching, AI use was significantly associated with an increased risk of depression compared to no use of AI (*p*=0.007, **Figure 4A**). However, after matching, there was no significant difference in the incidence of depression in the group administered AI compared with the group not administered AI (*p*=0.635, **Figure 4B**). Before matching, the incidence of depression (diagnosis + antidepressants) was also significantly higher in the patients administered AI than in those not administered AI (*p*=0.049, **Figure 4C**). However, after matching, there was no difference in the incidence of depression between the two groups (*p*=0.533; **Figure 4D**).

Clinical characteristics of patients with breast cancer according to receipt of aromatase inhibitors (AIs) was compared in **Supplementary Table 8**. Before matching, AI use was a significant risk factor for the depression (only diagnosis) (*p=*0.007). However, after matching in the univariate analysis of the matched cohort, AI use was not associated with depression (*p=*0.635). AI use was also not a significant risk factor for depression (diagnosis + antidepressants) after matching (*p=*0.534). In terms of suicidal attempt, we could not find any significant difference in every analysis related to endocrine regimens.

### Sensitivity analysis of risk of depression according to diagnostic type

Clinical characteristics of patients with DCIS was exhibited in **Supplementary Table 9**. Before and after matching, in the DCIS patients, there was no statistical difference between the two groups in depression (only diagnosis and diagnosis+anti-depressants), and suicide attempt (**Supplementary Fig. 2**). Regardless of matching or the diagnostic method used depression, there was no significant difference according to tamoxifen.

We also analyzed the incidence of depression in patients with invasive breast cancer treated with tamoxifen or AI (**Supplementary Table 10**; **Supplementary Fig 3**, **7**). There was no statistical difference in the occurrence of depression according to the administration of tamoxifen or AI, before or after matching (**Supplementary Tables 11**, **12**). In the Cox proportional hazard model before and after matching, tamoxifen use was not a significant risk factor for depression (**Supplementary Fig 4**, **5**, **7**). Before matching, AI use increased the incidence of depression compared with no use (*p*=0.012). However, after matching, there was no statistical significance with the occurrence of depression according to AI use. When analyzed as another endpoint, depression (diagnosis + anti-depressants), there was no significant association between AI use and the incidence of depression.

## Discussion

In our study, patients with breast cancer who were administered AI and those who were administered tamoxifen showed no statistical differences in both the risk of depression (diagnosis only and diagnosis + antidepressant) and suicide attempts compared with those who did not receive endocrine treatment. To our knowledge, this is the first large-scale nationwide longitudinal cohort study to analyze the risk of depression according to the endocrine treatment type. The strength of this study is that the average follow-up period was approximately 10 years, which is relatively long.

In hormone receptor-positive breast cancer, endocrine treatment, including tamoxifen and AI, is the standard treatment (5, 12). These treatments decrease recurrence and increase the survival rates of patients with breast cancer. Therefore, physicians should understand the potential adverse events of endocrine treatment and further explain its risks and benefits to patients. The NSABP P-1 trial was a pivotal trial evaluating the efficacy of tamoxifen, and the degree of depression caused by taking tamoxifen was analyzed using a questionnaire (13). The questionnaire survey was administered every six-months for a total of 3 years, and the degree of depression in both groups according to tamoxifen use showed no statistical difference (odds ratio=0.98, 95% CI: 0.93–1.02) (7). In another large retrospective study, there was no significant difference in the degree of depression in 2,943 patients administered tamoxifen (HR=1.1, 95% CI: 0.89–1.2) (14). The fundamental limitations of the above studies are as follows: the questionnaire itself only analyzed the degree of depression, not a diagnosis, and the follow-up period was relatively short. In addition, several studies have reported that endocrine treatment is associated with an increased incidence of mood disorders, including depression (6, 15, 16). Previous randomized controlled trials reported no difference in the incidence rates of mood disorders between tamoxifen and AI use (17–19),; however, there were no large-scale prospective studies comparing patients with and without AI. In this study, endocrine treatment in patients with breast cancer did not increase the incidence of depression, including suicide attempts, even in the long term. These findings provide strong evidence using real-world data that should alleviate excessive concern of the risk of depression from endocrine treatment.

Several possible mechanisms have been proposed for endocrine treatment affecting the occurrence of depression. Estrogen has been reported to be associated with the positive effects of serotonin and norepinephrine (20). Neurotransmitters, including serotonin and norepinephrine, are related to anxiety and depression and are the main targets of antidepressant treatments. Several studies have reported that estradiol is involved in the increase in serotonin receptor density and urine excretion of the primary metabolite of serotonin (21–23). Decreased neurotransmitter activity was a high-risk factor for the occurrence of depression symptoms, and estrogen supplementation improved depression symptoms in postmenopausal and postnatal women (20, 24, 25). For these reasons, many physicians have expressed concern about the relationship between endocrine treatment and depression. Our study presents additional evidence for reducing these concerns.

This study has several limitations. First, the accuracy of the depression diagnosis cannot be guaranteed because we utilized data from an insurance claims database. In general, a definite diagnosis of depression can be made by a trained psychiatrist according to the Diagnostic and Statistical Manual of Mental Disorders (DSM) 5^th^ edition criteria. Although we attempted to overcome this limitation by including only patients with both diagnostic codes of depression and antidepressant prescription records, this limitation may not be fully addressed. It was also not possible to determine the severity of depression using these data, but this could be assessed using several neuropsychological tests. To partially solve this problem, we attempted to include additional analyses using patients with suicide attempts. Second, there was no analysis of the risk of depression according to the endocrine treatment duration. This is because it was not possible to confirm medication adherence, even if the number of days for the drug prescription was given. Another limitation is that we observed the outcomes for almost 10 years; surviving patients with breast cancer might be particularly resilient to disease, even though we only included patients who underwent curative resection. Lastly, although our sample size was large, we utilized longitudinal data from the South Korean population, and the findings may not be generalizable to patients in other countries or patients of different ethnicities.

In this nationwide cohort study on breast cancer patients, endocrine treatment, including tamoxifen and AI, was not associated with the risk for depression before and after adjusting for age, chemotherapy, and comorbidities. The findings of our study support those produced by previous prospective randomized controlled trials demonstrating that there is no evidence that the endocrine treatment increases the risk of depression.

## Data availability statement

The original contributions presented in the study are included in the article/**Supplementary Material**. Further inquiries can be directed to the corresponding author.

## Ethics statement

The studies involving human participants were reviewed and approved by the Institutional Review Board (Local IRB number: 3-2021-0050) of Gangnam Severance Hospital. Written informed consent for participation was not required for this study in accordance with the national legislation and the institutional requirements.

## Author contributions

JO and CY had full access to all of the data in the study and takes responsibility for the integrity of the data and accuracy of the data analysis. Conceptualization: JO and CY. Data curation: HL, SJ. Funding acquisition: JO. Investigation: JO, HL, SJ and CY. Methodology: JO, HL, SJ and CY. Resources: HL, SJ. Formal analysis: JO and CY. Supervision, DK, JS, WP, and JK. Writing-original draft: JO and CY. All authors contributed to the article and approved the submitted version.

## Funding

This study was supported by the National Research Foundation of Korea (NRF) grant funded by the Korea government (MSIT) (No. 2020R1C1C1007440).

## Conflict of interest

The authors declare that the research was conducted in the absence of any commercial or financial relationships that could be construed as a potential conflict of interest.

## Publisher’s note

All claims expressed in this article are solely those of the authors and do not necessarily represent those of their affiliated organizations, or those of the publisher, the editors and the reviewers. Any product that may be evaluated in this article, or claim that may be made by its manufacturer, is not guaranteed or endorsed by the publisher.

## Abbreviations

AI, aromatase inhibitor; NSABP, National Surgical Adjuvant Breast and Bowel Project; DCIS, ductal carcinoma *in situ*; NHI, Korean National Health Insurance; HIRA, Health Insurance Review and Assessment Service; ICD-10, International Classification of Disease; 10th revision; IRB, Institutional Review Board; COPD, chronic obstructive pulmonary disease; CKD, chronic kidney disease; HR, hazard ratio; CI, confidence intervals; SD, standard deviation; DSM, Diagnostic and Statistical Manual of Mental Disorders.
