# Supplementary material for: Risk of developing depression from endocrine treatment: A nationwide cohort study of women administered treatment for breast cancer in South Korea
Source: Front Oncol. 2022 Sep 20;12:980197. doi: 10.3389/fonc.2022.980197 (PMC9530937; doi:10.3389/fonc.2022.980197)
Supplement: Supplementary file 1 [file DataSheet_1.docx]

**Supplementary Figure Legends**

**Supplementary Figure 1. Kaplan–Meier analysis of incidence of depression (depression + prescribed anti-depressants) in breast cancer patients according to endocrine treatment.** Before matching, there was no significant difference between the patients who underwent endocrine treatment and who did not (a, *p =* 0.590, log-rank test). After matching, there was still no significant difference between these two groups (b, *p* = 0.249).

**Supplementary Figure 2. Subgroup analysis of incidence of depression in patients with DCIS according to tamoxifen use.** Before matching, tamoxifen use was not significantly associated with an increased risk of depression (only diagnosis) in the patients administered tamoxifen than in those who were not administered tamoxifen (a, *p =* 0.579). After matching, there was still no significant difference between these two groups (b, *p* = 0.859). When the incidence of depression (diagnosis + anto-depressant) was analyzed in the patients, the patients administered tamoxifen did not show a signficantly higher risk of depression than those not administered tamoxifen (c, *p =* 0.527). After matching, there was still no significant difference between these two groups (d, *p* = 0.849).

**Supplementary Figure 3. Subgroup analysis of incidence of depression in patients with invasive breast cancer according to endocrine treatment.** Before matching, endocrine treatment was not significantly associated with an increased risk of depression (only diagnosis) compared with no endocrine treatment (a, *p =* 0.570). After matching, there was still no significant difference between these two groups (b, *p* = 0.252). When the incidence of depression (diagnosis + anto-depressant) was analyzed in the patients, endocrine treatment did not exhibit a signficantly higher risk of depression than no endocrine treatment (c, *p =* 0.414). After matching, the patients who underwent endocrine treatment had no signficant difference in the incidence of depression compared with those who did not undergo endocrine treatment (d, *p* = 0.238).

**Supplementary Figure 4. Subgroup analysis of incidence of depression in patients with invasive breast cancer according to tamoxifen use.** Before matching, tamoxifen use was not significantly associated with an increased risk of depression (only diagnosis) compared with no tamoxifen use (a, *p =* 0.320). After matching, there was still no significant difference (b, *p* = 0.854). When the incidence of depression (diagnosis + anto-depressant) was analyzed in the patients, the patients administered tamoxifen did not exhibit a signficantly higher risk of depression than those not administered tamoxifen (c, *p =* 0.181). After matching, there was still no significant difference between these two groups (d, *p* = 0.077).

**Supplementary Figure 5. Subgroup analysis of incidence of depression in patients with invasive breast cancer according to aromatase inhibitors.** Before matching, patients administered aromatase inhibitors exhibited a significantly increased risk of depression (only diagnosis) compared with those not administered aromatase inhibitors (a, *p* = 0.012). However, after matching, there was no significant difference between these two groups (b, *p* = 0.635). When the incidence of depression (diagnosis + anto-depressant) was analyzed in the patients, the patients administered aromatase inhibitors did not exhibit a signficantly higher risk of depression than those not administreed aromatase inhibitors (c, *p* = 0.061). After matching, there was no significant difference between these two groups (d, *p* = 0.533).

**Supplementary Figure 6. Forest plots on the risk of depression according to endocrine regimens.**

A: Depression in all patients (only diagnosis), B: Depression in all patients (diagnosis+anti-depressant), C: Depression in patients with receiving tamoxifen (only diagnosis), D: Depression in patients with receiving tamoxifen (diagnosis+anti-depressant), E: Depression in patients with receiving AI (only diagnosis), F: Depression in patients with receiving AI (diagnosis+anti-depressant)

**Supplementary Figure 7. Forest plots on the risk of depression according to diagnostic type.**

A: Depression in DCIS patients with receiving tamoxifen (only diagnosis), B: Depression in DCIS patients with receiving tamoxifen (diagnosis+anti-depressant), C: Depression in breast cancer patients (only diagnosis), ) D: Depression in breast cancer patients (diagnosis+anti-depressant), E: Depression in breast cancer patients with receiving tamoxifen (only diagnosis), F: Depression in breast cancer patients with receiving tamoxifen (diagnosis+anti-depressant), G: Depression in breast cancer patients with receiving AI (only diagnosis), H: Depression in breast cancer patients with receiving AI (diagnosis+anti-depressant)
