# Supplementary figures and images for: Risk of developing depression from endocrine treatment: A nationwide cohort study of women administered treatment for breast cancer in South Korea
Source: Front Oncol. 2022 Sep 20;12:980197. doi: 10.3389/fonc.2022.980197 (PMC9530937; doi:10.3389/fonc.2022.980197)

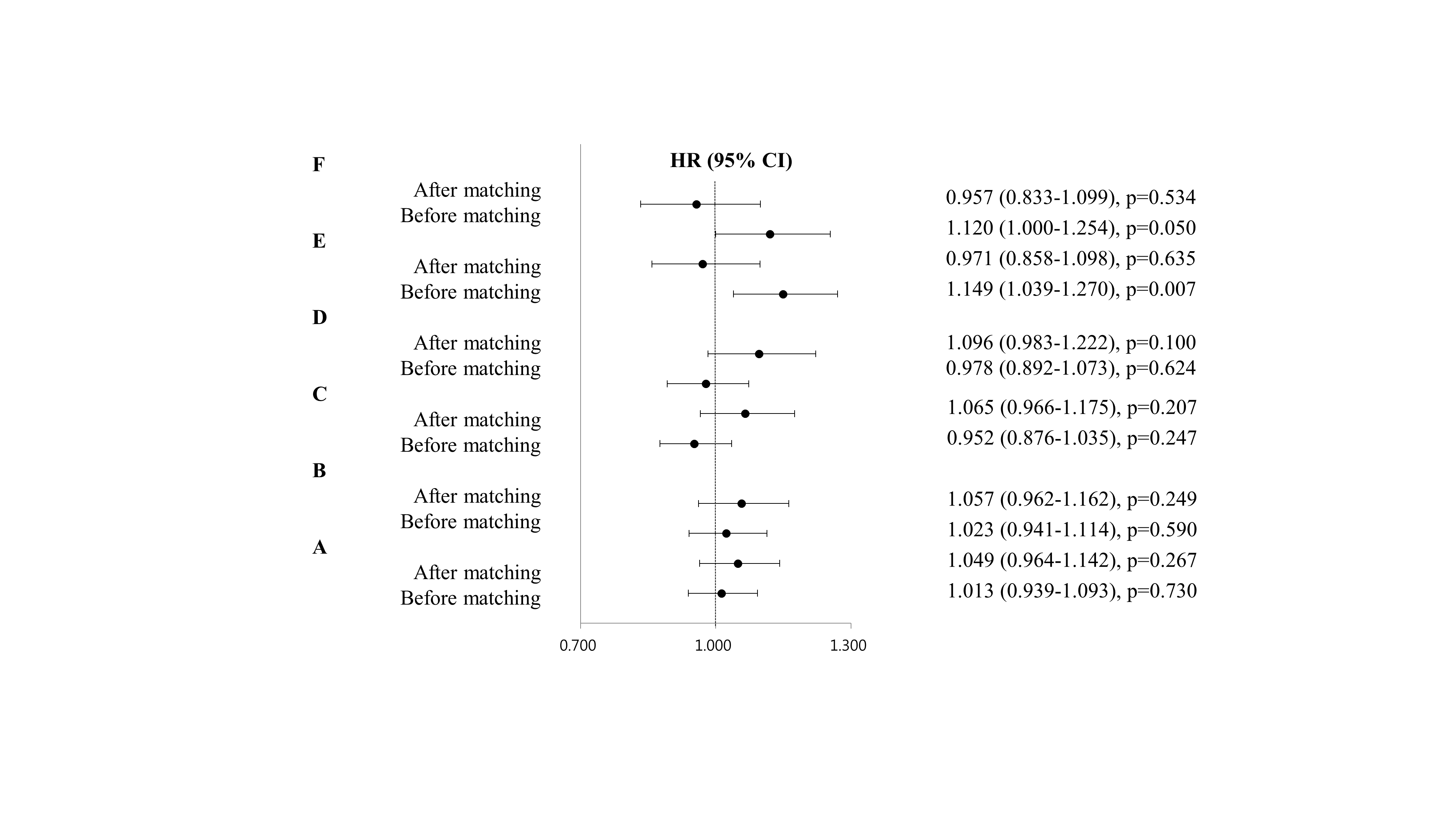

Supplement: Supplementary file 2 [file Image_1.tif]

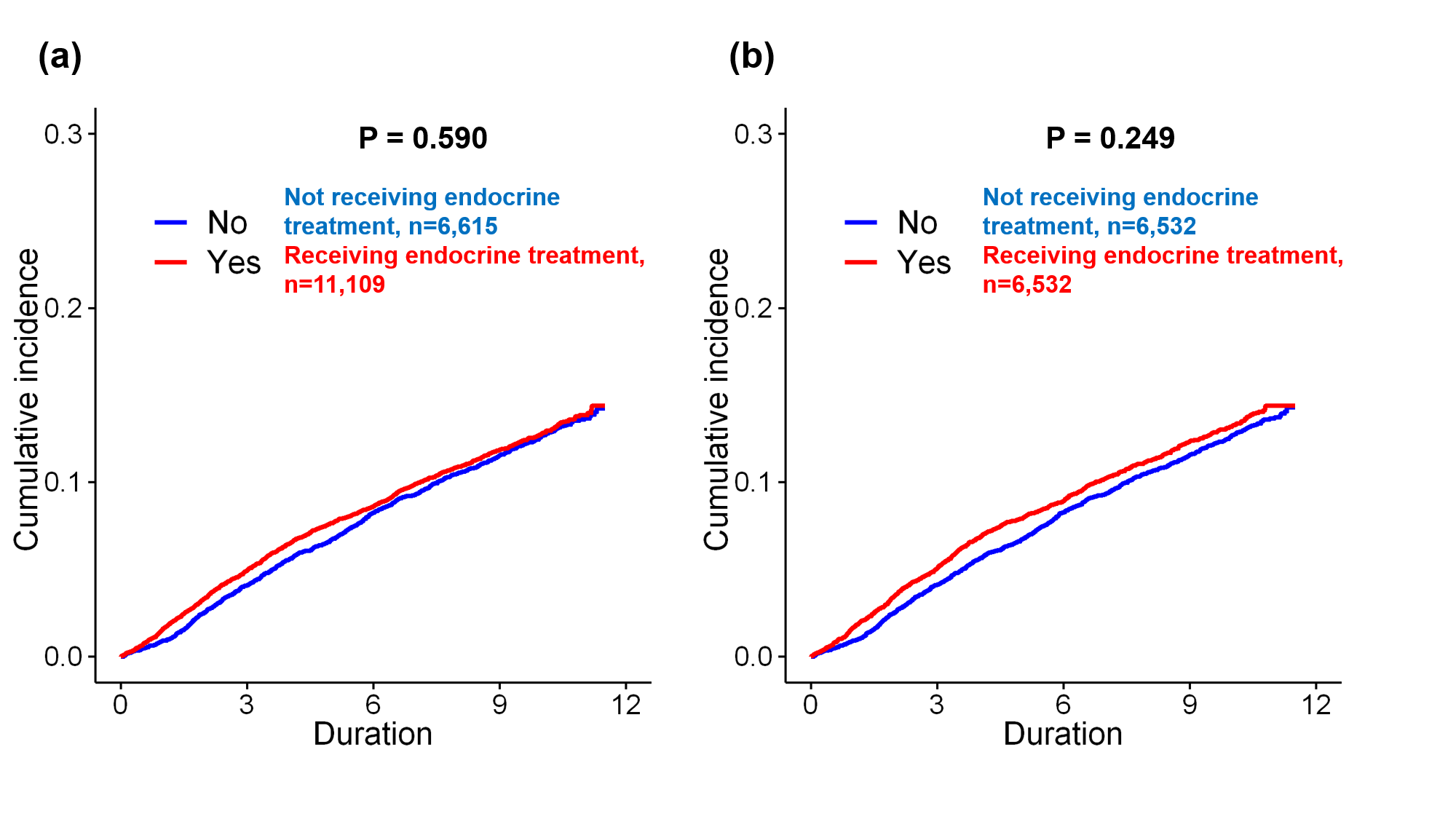

Supplement: Supplementary file 3 [file Image_2.tif]

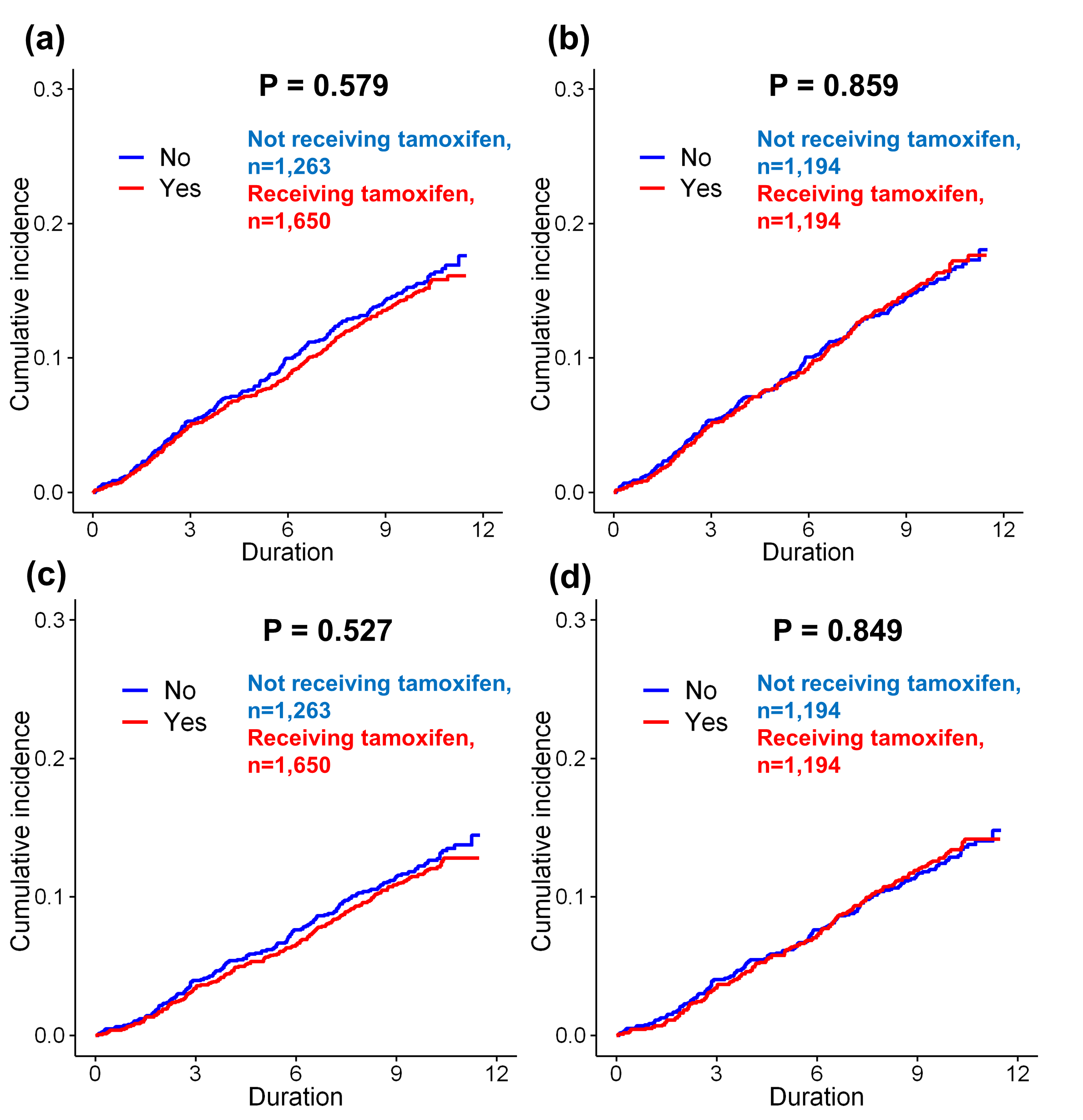

Supplement: Supplementary file 4 [file Image_3.tif]

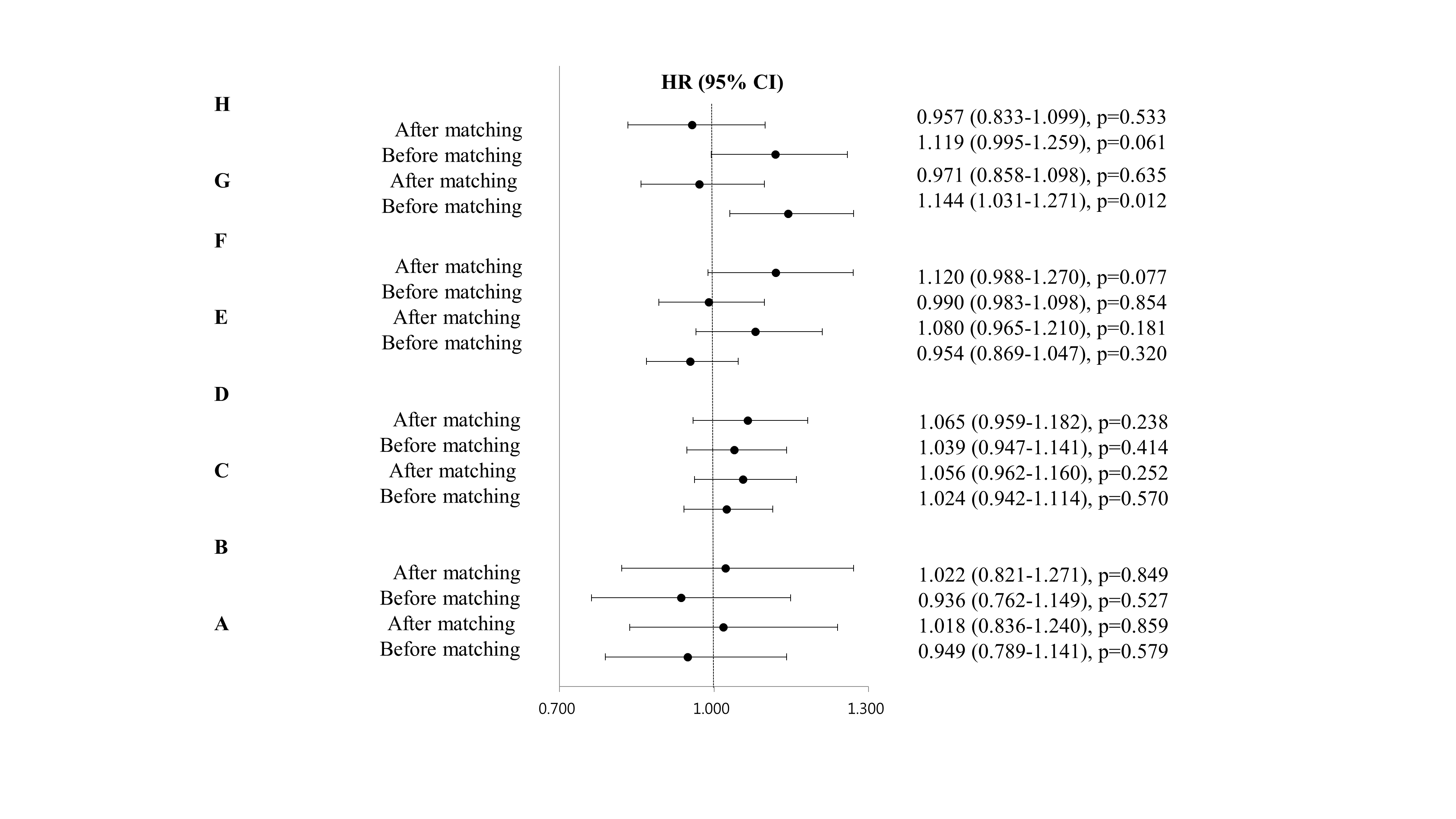

Supplement: Supplementary file 5 [file Image_4.tif]

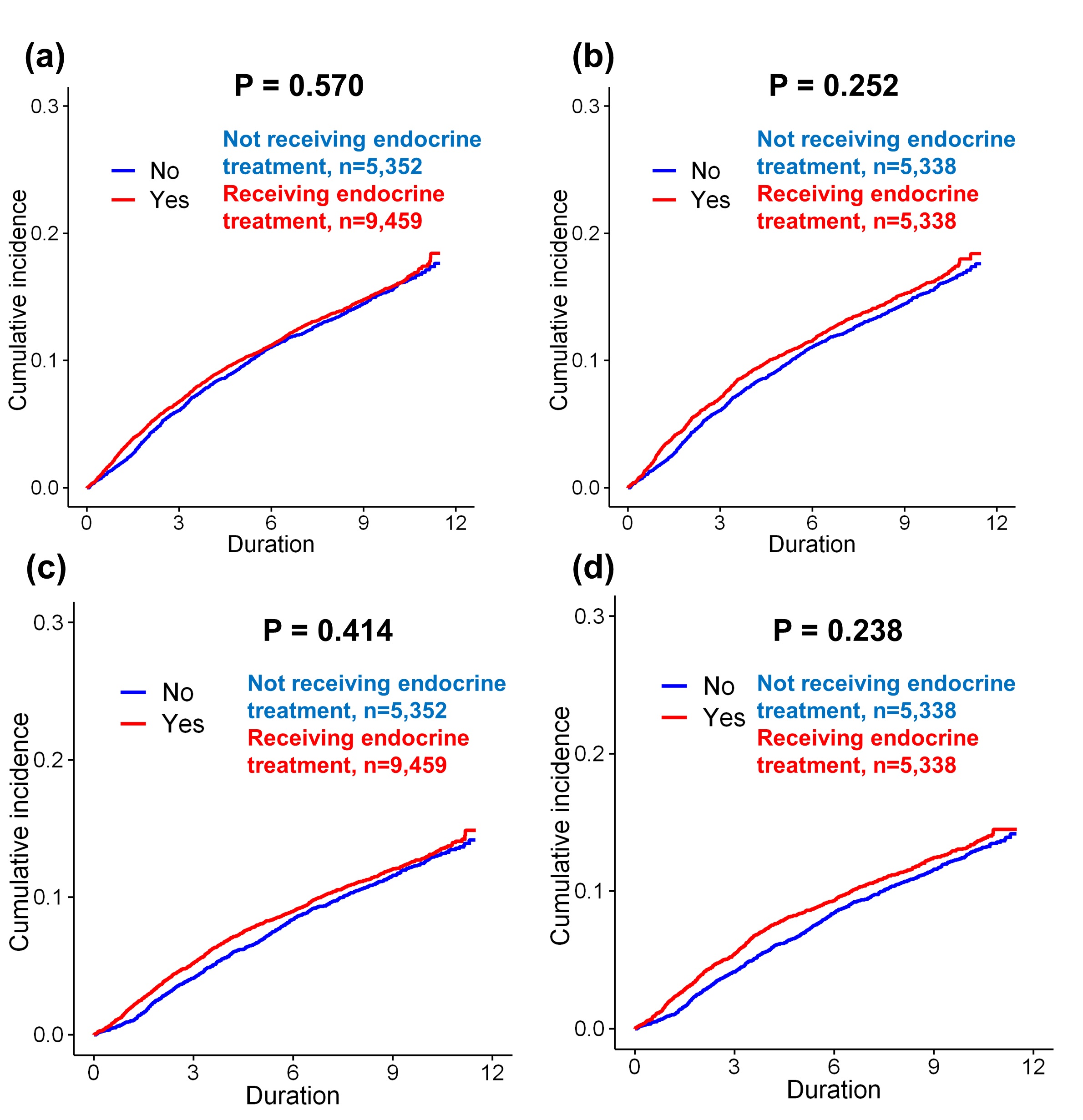

Supplement: Supplementary file 6 [file Image_5.tif]

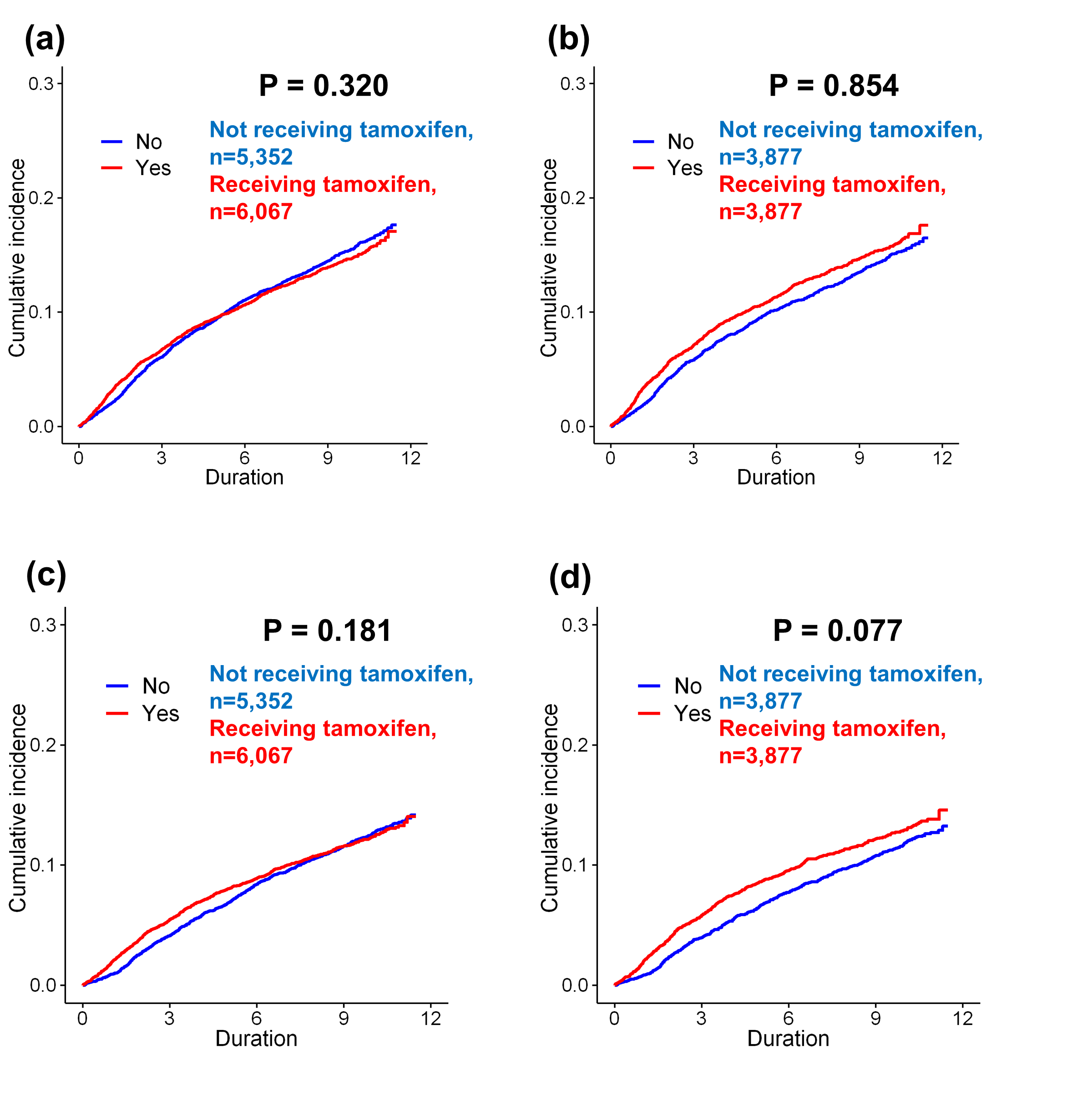

Supplement: Supplementary file 7 [file Image_6.tif]

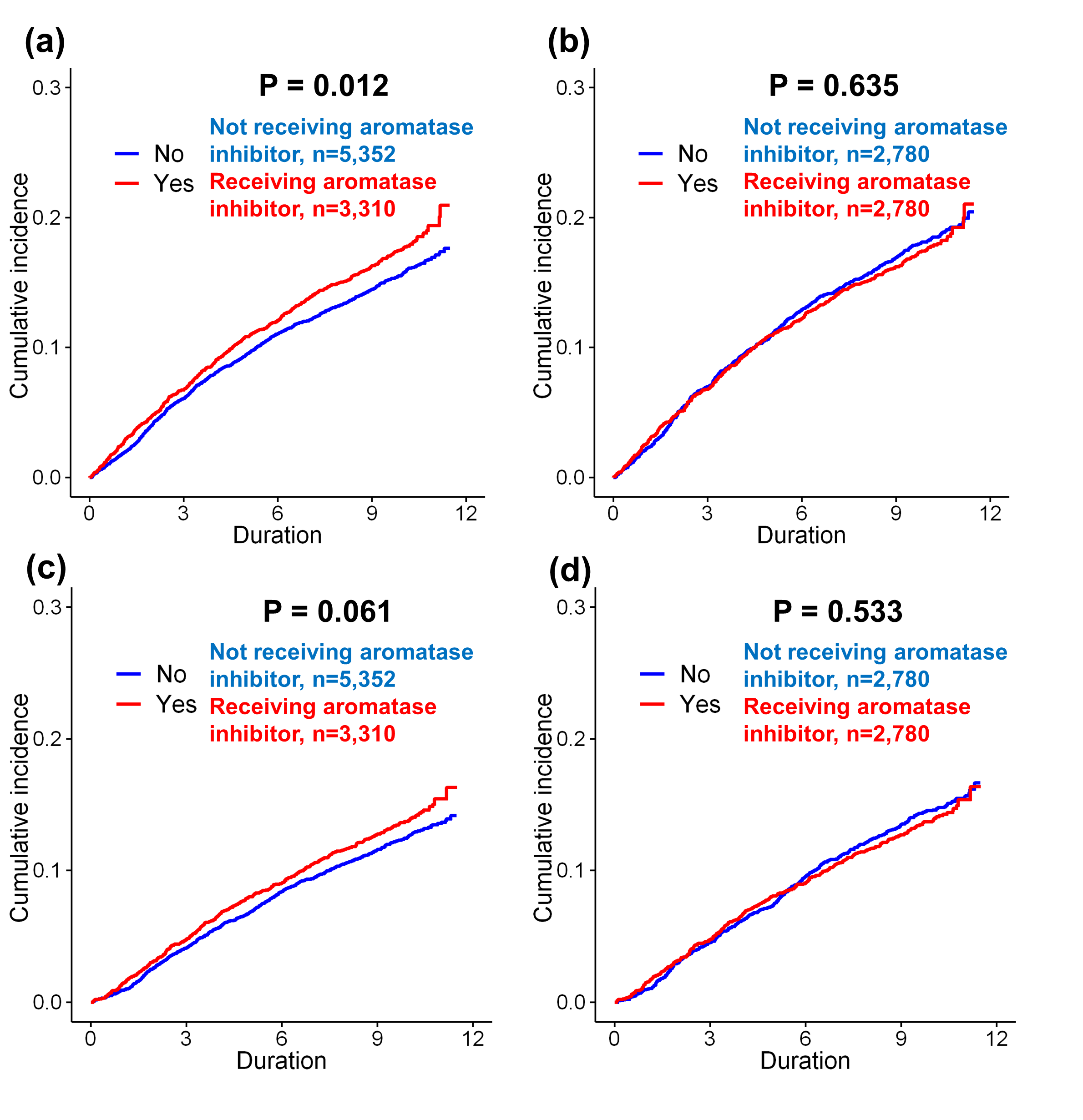

Supplement: Supplementary file 8 [file Image_7.tif]
