# Supplementary material for: Risk of developing depression from endocrine treatment: A nationwide cohort study of women administered treatment for breast cancer in South Korea
Source: Front Oncol. 2022 Sep 20;12:980197. doi: 10.3389/fonc.2022.980197 (PMC9530937; doi:10.3389/fonc.2022.980197)
Supplement: Supplementary file 9 [file Table_1.docx]

**Supplementary Table 1. Behavior surgical code for breast and axillary surgery**

| **Behavior code** | **ICD-10 Code** |
| --- | --- |
| N7133 | wide excision |
| N7134 | wide excision of axillary breast |
| N7136 | wide excision with axillary surgery |
| N7137 | wide excision without axillary surgery |
| N7138 | total mastectomy with axillary surgery |
| N7139 | total mastectomy without axillary surgery |

**Supplementary Table 2. Diagnostic codes of intentional self-harm and probable suicide attempts**

| **Diagnostic codes** | **ICD-10 code** |
| --- | --- |
| **Intentional self-harm** | X60-X85 |
| **Probable suicide attempts** |  |
| wrist laceration | S61.9 |
| poisoning by psychotropic drugs | T43 |
| acute drug intoxication | T50.9 |
| toxic effects of organic solvents, corrosive substances, carbon monoxide, pesticides, other specified substances, and unspecified substances | T52, T54, T58, T60, and T65 |
| handgun, rifle, or other firearm discharges | W32, W33, and W34 |
| accidental suffocation and strangulation in bed | W75 |
| other accidental hanging and strangulation | W76 |
| event of undermined intent | Y10-Y34 |
| asphyxia | R09 |
| falls | W13-W19 |

**Supplementary Table 3. Anti-depressant and anti-anxiety drug prescription codes**

| **Drugs** | ***Code** |
| --- | --- |
| **Anti-depressant** |  |
| Escitalopram | 474803ATB, 474802ATB, 474801ATB, 474804ATB, 521102ATD, 521101ATD |
| Vortioxetine | 628502ATB, 628504ATB, 628501ATB, 628503ATB |
| Sertraline | 227001ATB, 227002ATB, 227003ATB |
| Fluoxetine | 161502ACH, 161501ACH, 161502ATB, 161501ATB, 161502ATD, 161504ACR |
| Paroxetine | 209301ATB, 209302ATB, 209304ATR, 209305ATR, 209306ATR |
| Fluvoxamine | 162501ATB, 162502ATB |
| Mirtazapine | 196202ATD, 196201ATD, 196201ATB, 196202ATB, 196204ATD, 196204ATB |
| Venlafaxine | 247504ACR, 247502ACR, 247504ATR, 247502ATR |
| Desvenlafaxine | 626402ATR, 626401ATR |
| Duloxetine | 495502ACE, 495501ACE, 495501ATE, 495502ATE |
| Milnacipran | 355801ACH, 355802ACH, 355803ACH |
| Tianeptine | 229601ATB |
| Imipramine | 173701ATB |
| Nortriptyline | 203401ATB, 203402ATB |
| Doxepin | 149204ATB, 451901CCM, 149203ATB |
| Amitriptyline | 107501ATB, 107502ATB, 480600ATB, 513000ATB, 107504ATB, |
| Agomelatine | 613101ATB |
| **Anti-anxiety** |  |
| Alprazolam | 105502ATB, 105504ATB, 105505ATB, 105501ATB, |
| Clonazepam | 136401ATB |
| Lorazepam | 185504ATB, 185501ATB |
| Diazepam | 142902ATB, 142903ATB |
| Etizolam | 156501ATB, 156503ATB, 156502ATB |
| Chlordiazepoxide | 131202ATB, 131201ATB |
| Clobazam | 135702ATB |

**Supplementary Table 4. Anti-hormonal drug prescription codes**

| **Drugs** | ***Code** |
| --- | --- |
| **Tamoxifen** | 234501ATB, 234502ATB |
| **Aromatase inhibitor** |  |
| Letrozole | 182201ATB |
| Anastrozole | 109001ATB |
| Exemestane | 358401ATB |

*Drug claim code at NHI

**Supplementary Table 5. Chemotherapy drug prescription codes**

| **Drugs** | ***Code** |
| --- | --- |
| **Taxane** | 148301BIJ, 148302BIJ, 148303BIJ, 148304BIJ, 148305BIJ, 148306BIJ, 148309BIJ, 148310BIJ, 148338BIJ, 148339BIJ, 148340BIJ, 148341BIJ, 148342BIJ, 148344BIJ, 148345BIJ, 148346BIJ, 148348BIJ, 148349BIJ, 148350BIJ, 148351BIJ |
| **Doxorubicin** | 149401BIJ, 149402BIJ, 149403BIJ, 149404BIJ, 149405BIJ, 149406BIJ, 149430BIJ, 149431BIJ, 149432BIJ, 149433BIJ, 149434BIJ, 149435BIJ |
| **Cyclophosphamide** | 139001ATB, 139003BIJ, 139004BIJ, 139005BIJ |
| **Fluorouracil** | 161401BIJ, 161402BIJ, 161404BIJ, 161430BIJ, 161431BIJ, 161432BIJ |
| **Methotrexate** | 192101ATB, 192102BIJ, 192103BIJ, 192104BIJ, 192105BIJ, 192107ATB, 192107BIJ, 192108BIJ, 192109BIJ, 192110BIJ, 192111BIJ, 192132BIJ, 192134BIJ, 192136BIJ, 192138BIJ, 192139BIJ, 192140BIJ, 192141BIJ, 192142BIJ, 192143BIJ, 192144BIJ |

*Drug claim code at NHI

**Supplementary Table 6. Synthroid and steroid drug prescription codes**

| **Drugs** | ***Code** |
| --- | --- |
| **Synthroid** | 183601ATB, 183608ATB, 183604ATB, 183602ATB, 183606ATB, 183610ATB, 183611ATB, 183607ATB, 183603ATB, 183609ATB |
| **Steroid** |  |
| Methylprednisolone | 193302ATB, 217001ATB, 193604BIJ, 193601BIJ, 193530BIJ, 217302BIJ, 193603BIJ |
| Hydrocortisone | 170901ATB, 170906ATB, 171201BIJ, 171202BIJ, 243202ATB |
| Triamcinolone | 243203ATB, 243201ATB, 243336BIJ, 243303BIJ, 243335BIJ, 243337BIJ, 243301BIJ, 243305BIJ |
| Betamethasone | 116502BIJ, 116530BIJ, 116401ATB |
| Dexamethasone | 142201BIJ, 142232BIJ, 141901ATB, 141903ATB, 141904ATB |

*Drug claim code at NHI

**Supplementary Table 7. Comparison of clinical characteristics of patients according to taking tamoxifen**

|  | **Before matching** |  |  | **After matching** |  |  |
| --- | --- | --- | --- | --- | --- | --- |
|  | **Patients not receiving tamoxifen, n=6,615 (%)** | **Patients receiving tamoxifen, n=7,717 (%)** | ***P* value** | **Patients not receiving tamoxifen, n=5,071 (%)** | **Patients receiving tamoxifen, n=5,071 (%)** | ***P* value** |
| **Depression (only diagnosis)** |  |  | 0.061 |  |  | 0.513 |
| **No** | 5,541 (83.8) | 6,552 (84.9) |  | 4,285 (84.5) | 4,261 (84.0) |  |
| **Yes** | 1,074 (16.2) | 1,165 (15.1) |  | 786 (15.5) | 810 (16.0) |  |
| **Depression (diagnosis + anti-depressant)** |  |  | 0.246 |  |  | 0.298 |
| **No** | 5,751 (86.9) | 6,759 (87.6) |  | 4,439 (87.5) | 4,404 (86.9) |  |
| **Yes** | 864 (13.1) | 958 (12.4) |  | 632 (12.5) | 667 (13.2) |  |
| **Depression (only diagnosis) + suicidal attempt** |  |  | 0.662 |  |  | 0.853 |
| **No** | 6,599 (99.8) | 7,701 (99.8) |  | 5,056 (99.7) | 5,057 (99.7) |  |
| **Yes** | 16 (0.2) | 16 (0.2) |  | 15 (0.3) | 14 (0.3) |  |
| **Depression (diagnosis + anti-depressant) + suicidal attempt** |  |  | 0.431 |  |  | 0.548 |
| **No** | 6,600 (99.8) | 7,704 (99.8) |  | 5,057 (99.7) | 5,060 (99.8) |  |
| **Yes** | 15 (0.2) | 13 (0.2) |  | 14 (0.3) | 11 (0.2) |  |
| **Suicidal attempt** |  |  | 0.677 |  |  | 0.439 |
| **No** | 6,509 (98.4) | 7,600 (98.5) |  | 4,991 (98.4) | 4,981 (98.2) |  |
| **Yes** | 106 (1.6) | 117 (1.5) |  | 80 (1.6) | 90 (1.8) |  |
| **Endocrine disorder (including thyroid disease)** |  |  | 0.055 |  |  | 0.203 |
| **No** | 6,028 (91.1) | 7,101 (92.0) |  | 4,678 (92.3) | 4,643 (91.6) |  |
| **Yes** | 587 (8.9) | 616 (8.0) |  | 393 (7.8) | 428 (8.4) |  |
| **Taking synthroid** |  |  | 0.301 |  |  | 0.357 |
| **No** | 6,415 (97.0) | 7,506 (97.3) |  | 4,942 (97.5) | 4,927 (97.2) |  |
| **Yes** | 200 (3.0) | 211 (2.7) |  | 129 (2.5) | 144 (2.8) |  |
| **Taking steroids** |  |  | 0.007 |  |  | 0.816 |
| **No** | 2,135 (32.3) | 2,654 (34.4) |  | 1,660 (32.7) | 1,649 (32.5) |  |
| **Yes** | 4,480 (67.7) | 5,063 (65.6) |  | 3,411 (67.3) | 3,422(67.5) |  |
| **Diabetes** |  |  | <0.001 |  |  | 0.860 |
| **No** | 5,834 (88.2) | 7,155 (92.7) |  | 4,633 (91.4) | 4,628 (91.3) |  |
| **Yes** | 781 (11.8) | 562 (7.3) |  | 438 (8.6) | 443 (8.7) |  |
| **Hypertension** |  |  | <0.001 |  |  | 0.426 |
| **No** | 5,100 (77.1) | 6,594 (85.5) |  | 4,234 (83.5) | 4,204 (82.9) |  |
| **Yes** | 1,515 (22.9) | 1,123 (14.6) |  | 837 (16.5) | 867 (17.1) |  |
| **Hyperlipidemia** |  |  | <0.001 |  |  | 0.801 |
| **No** | 5,400 (81.6) | 6,785 (87.9) |  | 4,329 (85.4) | 4,320 (85.2) |  |
| **Yes** | 1,215 (18.4) | 932 (12.1) |  | 742 (14.6) | 751 (14.8) |  |
| **COPD** |  |  | <0.001 |  |  | 0.581 |
| **No** | 6,385 (96.5) | 7,540 (97.7) |  | 4,930 (97.2) | 4,939 (97.4) |  |
| **Yes** | 230 (3.5) | 177 (2.3) |  | 141 (2.8) | 132 (2.6) |  |
| **CKD** |  |  | 0.008 |  |  | 0.159 |
| **No** | 6,576 (99.4) | 7,694 (99.7) |  | 5,046 (99.51 | 5,055 (99.7) |  |
| **Yes** | 39 (0.6) | 23 (0.3) |  | 25 (0.5) | 16 (0.3) |  |
| **LC** |  |  | 0.082 |  |  | 0.683 |
| **No** | 6,590 (99.6) | 7,700 (99.8) |  | 5,058 (99.7) | 5,060 (99.8) |  |
| **Yes** | 25 (0.4) | 17 (0.2) |  | 13 (0.3) | 11 (0.2) |  |
| **Heart failure** |  |  | <0.001 |  |  | 0.337 |
| **No** | 6,557 (99.1) | 7,684 (99.6) |  | 5,032 (99.2) | 5,040 (99.4) |  |
| **Yes** | 58 (0.9) | 33 (0.4) |  | 39 (0.8) | 31 (0.6) |  |
| **Chemotherapy** |  |  | 0.088 |  |  | 0.905 |
| **Not done** | 2,912 (44.0) | 3,507 (45.5) |  | 2,412 (47.6) | 2,406 (47.5) |  |
| **Done** | 3,703 (55.6) | 4,210 (54.6) |  | 2,659 (52.4) | 2,665 (52.6) |  |
| **Age (year, mean±SD)** | 50.83±11.29 | 46.42±8.64 | <0.001 | 47.72±9.97 | 47.67±9.84 | 0.788 |

COPD, chronic obstruction pulmonary disease; CKD, chronic kidney disease; LC, liver cirrhosis; SD, standard deviation

**Supplementary Table 8. Comparison of clinical characteristics of patients with breast cancer according to receipt of aromatase inhibitors (AIs)**

|  | **Before matching** |  |  | **After matching** |  |  |
| --- | --- | --- | --- | --- | --- | --- |
|  | **Patients not receiving AIs, n=6,615 (%)** | **Patients receiving AIs, n=3,310 (%)** | ***P* value** | **Patients not receiving AIs, n=2,780 (%)** | **Patients receiving AI, n=2,780 (%)** | ***P* value** |
| **Depression (only diagnosis)** |  |  | 0.046 |  |  | 0.314 |
| **No** | 5,541 (83.8) | 2,720 (82.2) |  | 2,258 (81.2) | 2,287 (82.3) |  |
| **Yes** | 1,074 (16.2) | 590 (17.8) |  | 522 (18.8) | 493 (17.7) |  |
| **Depression (diagnosis + anti-depressant)** |  |  | 0.187 |  |  | 0.254 |
| **No** | 5,751 (86.9) | 2,846 (86.0) |  | 2,361 (84.9) | 2,391 (86.0) |  |
| **Yes** | 864 (13.1) | 464 (14.0) |  | 419 (15.1) | 389 (14.0) |  |
| **Depression (only diagnosis) + suicidal attempt** |  |  | 0.104 |  |  | 0.058 |
| **No** | 6,599 (99.8) | 3,307 (99.9) |  | 2,772 (99.7) | 2,778 (99.9) |  |
| **Yes** | 16 (0.2) | 3 (0.1) |  | 8 (0.3) | 2 (0.1) |  |
| **Depression (diagnosis + anti-depressant) + suicidal attempt** |  |  | 0.133 |  |  | 0.058 |
| **No** | 6,600 (99.8) | 3,307 (99.9) |  | 2,772 (99.7) | 2,778 (99.9) |  |
| **Yes** | 15 (0.2) | 3 (0.1) |  | 8 (0.3) | 2 (0.1) |  |
| **Suicidal attempt** |  |  | 0.487 |  |  | >0.999 |
| **No** | 6,509 (98.4) | 3,263 (98.6) |  | 2,741 (98.6) | 2,741 (98.6) |  |
| **Yes** | 106 (1.6) | 47 (1.4) |  | 39 (1.4) | 39 (1.4) |  |
| **Endocrine disorder (including thyroid disease)** |  |  | 0.949 |  |  | 0.778 |
| **No** | 6,028 (91.1) | 3,015 (91.1) |  | 2,528 (90.9) | 2,534 (91.2) |  |
| **Yes** | 587 (8.9) | 295 (8.9) |  | 252 (9.1) | 246 (8.9) |  |
| **Taking synthroid** |  |  | 0.332 |  |  | 0.714 |
| **No** | 6,415 (97.0) | 3,198 (96.6) |  | 2,681 (96.4) | 2,686 (96.6) |  |
| **Yes** | 200 (3.0) | 112 (3.4) |  | 99 (3.6) | 94 (3.4) |  |
| **Taking steroids** |  |  | 0.011 |  |  | 0.977 |
| **No** | 2,135 (32.3) | 985 (29.8) |  | 829 (29.8) | 828 (29.8) |  |
| **Yes** | 4,480 (67.7) | 2,325 (70.2) |  | 1,951 (70.2) | 1,952 (70.2) |  |
| **Diabetes** |  |  | <.001 |  |  | 0.890 |
| **No** | 5,834 (88.2) | 2,688 (81.2) |  | 2,274 (81.8) | 2,270 (81.7) |  |
| **Yes** | 781 (11.8) | 622 (18.8) |  | 506 (18.2) | 510 (18.4) |  |
| **Hypertension** |  |  | <0.001 |  |  | 0.526 |
| **No** | 5,100 (77.1) | 1,965 (59.4) |  | 1,730 (62.2) | 1,707 (61.4) |  |
| **Yes** | 1,515 (22.9) | 1,345 (40.6) |  | 1,050 (37.8) | 1,073 (38.6) |  |
| **Hyperlipidemia** |  |  | <0.001 |  |  | 0.904 |
| **No** | 5,400 (81.6) | 2,347 (70.9) |  | 2,016 (72.5) | 2,020 (72.7) |  |
| **Yes** | 1,215 (18.4) | 963 (29.1) |  | 764 (27.5) | 760 (27.3) |  |
| **COPD** |  |  | <0.001 |  |  | 0.284 |
| **No** | 6,385 (96.5) | 3,111 (94.0) |  | 2,640 (95.0) | 2,622 (94.3) |  |
| **Yes** | 230 (3.5) | 199 (6.0) |  | 140 (5.0) | 158 (5.7) |  |
| **CKD** |  |  | 0.384 |  |  | 0.302 |
| **No** | 6,576 (99.4) | 3,295 (99.6) |  | 2,760 (99.3) | 2,766 (99.5) |  |
| **Yes** | 39 (0.6) | 15 (0.5) |  | 20 (0.7) | 14 (0.5) |  |
| **LC** |  |  | 0.114 |  |  | 0.723 |
| **No** | 6,590 (99.6) | 3,290 (99.4) |  | 2,765 (99.5) | 2,763 (99.4) |  |
| **Yes** | 25 (0.4) | 20 (0.6) |  | 15 (0.5) | 17 (0.6) |  |
| **Heart failure** |  |  | 0.766 |  |  | 0.222 |
| **No** | 6,557 (99.1) | 3,279 (99.1) |  | 2,741 (98.6) | 2,751 (99.0) |  |
| **Yes** | 58 (0.9) | 31 (0.9) |  | 39 (1.4) | 29 (1.0) |  |
| **Chemotherapy** |  |  | <0.001 |  |  | 0.451 |
| **Not done** | 2,912 (44.0) | 1,226 (37.0) |  | 991 (35.7) | 1,018 (36.6) |  |
| **Done** | 3,703 (56.0) | 2,084 (63.0) |  | 1,789 (64.4) | 1,762 (63.4) |  |
| **Age (year, mean±SD)** | 50.83±11.29 | 59.28±7.97 | <0.001 | 58.76±8.274 | 58.76±8.22 | 0.991 |

COPD, chronic obstruction pulmonary disease; CKD, chronic kidney disease; LC, liver cirrhosis; SD, standard deviation

**Supplementary Table 9. Comparison of clinical characteristics of patients with DCIS**

|  | **Before matching** |  |  | **After matching** |  |  |
| --- | --- | --- | --- | --- | --- | --- |
|  | **Patients not receiving tamoxifen, n=1,263 (%)** | **Patients receiving tamoxifen, n=1,650 (%)** | ***P* value** | **Patients not receiving tamoxifen, n=1,194 (%)** | **Patients receiving tamoxifen, n=1,194 (%)** | ***P* value** |
| **Depression (only diagnosis)** |  |  | 0.518 |  |  | 0.956 |
| **No** | 1,059 (83.9) | 1,398 (84.7) |  | 997 (83.5) | 996 (83.4) |  |
| **Yes** | 204 (16.2) | 252 (15.3) |  | 197 (16.5) | 198 (16.6) |  |
| **Depression (diagnosis + anti-depressant)** |  |  | 0.468 |  |  | 0.952 |
| **No** | 1,097 (86.9) | 1,448 (87.8) |  | 1,034 (86.6) | 1,033 (86.5) |  |
| **Yes** | 166 (13.1) | 202 (12.2) |  | 160 (13.4) | 161 (13.5) |  |
| **Depression (only diagnosis) + suicidal attempt** |  |  | 0.740 |  |  | 0.726 |
| **No** | 1,260 (99.8) | 1,644 (99.6) |  | 1,191 (99.8) | 1,189 (99.6) |  |
| **Yes** | 3 (0.2) | 6 (0.4) |  | 3 (0.3) | 5 (0.4) |  |
| **Depression (diagnosis + anti-depressant) + suicidal attempt** |  |  | 0.706 |  |  | 0.687 |
| **No** | 1,261 (99.8) | 1,645 (99.7) |  | 1,192 (99.8) | 1,190 (99.7) |  |
| **Yes** | 2 (0.2) | 5 (0.3) |  | 2 (0.2) | 4 (0.3) |  |
| **Suicidal attempt** |  |  | 0.520 |  |  | 0.761 |
| **No** | 1,240 (98.2) | 1,625 (98.5) |  | 1,171 (98.1) | 1,173 (98.2) |  |
| **Yes** | 23 (1.8) | 25 (1.5) |  | 23 (1.9) | 21 (1.8) |  |
| **Endocrine disorder (including thyroid disease)** |  |  | 0.594 |  |  | 0.780 |
| **No** | 1,144 (90.6) | 1,504 (91.2) |  | 1,083 (90.7) | 1,079 (90.4) |  |
| **Yes** | 119 (9.4) | 146 (8.9) |  | 111 (9.3) | 115 (9.6) |  |
| **Taking synthroid** |  |  | 0.922 |  |  | 0.490 |
| **No** | 1,224 (96.9) | 1,598 (96.9) |  | 1,158 (97.0) | 1,152 (96.5) |  |
| **Yes** | 39 (3.1) | 52 (3.2) |  | 36 (3.0) | 42 (3.5) |  |
| **Taking steroids** |  |  | 0.050 |  |  | >0.999 |
| **No** | 410 (32.5) | 593 (35.9) |  | 391 (32.8) | 391 (32.8) |  |
| **Yes** | 853 (67.5) | 1,057 (64.1) |  | 803 (67.3) | 803 (67.3) |  |
| **Diabetes** |  |  | 0.006 |  |  | 0.773 |
| **No** | 1,133 (89.7) | 1,528 (92.6) |  | 1,091 (91.4) | 1,087 (91.0) |  |
| **Yes** | 130 (10.3) | 122 (7.4) |  | 103 (8.6) | 107 (9.0) |  |
| **Hypertension** |  |  | 0.268 |  |  | 0.643 |
| **No** | 1,005 (79.6) | 1,340 (81.2) |  | 955 (80.0) | 964 (80.7) |  |
| **Yes** | 258 (20.4) | 310 (18.8) |  | 239 (20.0) | 230 (19.3) |  |
| **Hyperlipidemia** |  |  | <0.001 |  |  | 0.789 |
| **No** | 1,019 (80.7) | 1,408 (85.3) |  | 980 (82.1) | 985 (82.5) |  |
| **Yes** | 244 (19.3) | 242 (14.7) |  | 214 (17.9) | 209 (17.5) |  |
| **COPD** |  |  | 0.129 |  |  | 0.495 |
| **No** | 1,225 (97.0) | 1,615 (97.9) |  | 1,164 (97.5) | 1,169 (97.9) |  |
| **Yes** | 38 (3.0) | 35 (2.1) |  | 30 (2.5) | 25 (2.1) |  |
| **CKD** |  |  | 0.075 |  |  | 0.108 |
| **No** | 1,252 (99.1) | 1,644 (99.6) |  | 1,184 (99.2) | 1,190 (99.7) |  |
| **Yes** | 11 (0.9) | 6 (0.4) |  | 10 (0.8) | 4 (0.3) |  |
| **LC** |  |  | 0.226 |  |  | 0.726 |
| **No** | 1,256 (99.5) | 1,646 (99.8) |  | 1,189 (99.6) | 1,191 (99.8) |  |
| **Yes** | 7 (0.6) | 4 (0.2) |  | 5 (0.4) | 3 (0.3) |  |
| **Heart failure** |  |  | 0.548 |  |  | 0.818 |
| **No** | 1,253 (99.2) | 1,640 (99.4) |  | 1,184 (99.2) | 1,185 (99.3) |  |
| **Yes** | 10 (0.8) | 10 (0.6) |  | 10 (0.8) | 9 (0.8) |  |
| **Chemotherapy** |  |  | 0.003 |  |  | 0.895 |
| **Not done** | 1,112 (88.0) | 1,508 (91.4) |  | 1,067 (89.4) | 1,065 (89.2) |  |
| **Done** | 151 (12.0) | 142 (8.6) |  | 127 (10.6) | 129 (10.8) |  |
| **Age (year, mean±SD)** | 49.81±10.29 | 48.11±9.15 | <0.001 | 49.21±9.83 | 49.11±9.60 | 0.805 |

COPD, chronic obstruction pulmonary disease; CKD, chronic kidney disease; LC, liver cirrhosis; SD, standard deviation

**Supplementary Table 10. Comparison of clinical characteristics of patients with invasive breast cancer according to endocrine treatment**

|  | **Before matching** |  |  | **After matching** |  |  |
| --- | --- | --- | --- | --- | --- | --- |
|  | **Patients not receiving endocrine treatment, n=5,352 (%)** | **Patients receiving endocrine treatment, n=9,459 (%)** | ***P* value** | **Patients not receiving endocrine treatment, n=5,338 (%)** | **Patients receiving endocrine treatment, n=5,338 (%)** | ***P* value** |
| **Depression (only diagnosis)** |  |  | 0.780 |  |  | 0.695 |
| **No** | 4,482 (83.7) | 7,938 (83.9) |  | 4,471 (83.8) | 4,456 (83.5) |  |
| **Yes** | 870 (16.3) | 1,521 (16.1) |  | 867 (16.2) | 882 (16.5) |  |
| **Depression (diagnosis + anti-depressant)** |  |  | 0.980 |  |  | 0.627 |
| **No** | 4,654 (87.0) | 8,224 (86.9) |  | 4,642 (87.0) | 4,625 (86.6) |  |
| **Yes** | 698 (13.0) | 1,235 (13.1) |  | 696 (13.0) | 713 (13.4) |  |
| **Depression (only diagnosis) + suicidal attempt** |  |  | 0.256 |  |  | 0.683 |
| **No** | 5,339 (99.8) | 9,444 (99.8) |  | 5,325 (99.8) | 5,327 (99.8) |  |
| **Yes** | 13 (0.2) | 15 (0.2) |  | 13 (0.2) | 11 (0.2) |  |
| **Depression (diagnosis + anti-depressant) + suicidal attempt** |  |  | 0.141 |  |  | 0.393 |
| **No** | 5,339 (99.8) | 9,446 (99.9) |  | 5,325 (99.8) | 5,329 (99.8) |  |
| **Yes** | 13 (0.2) | 13 (0.1) |  | 13 (0.2) | 9 (0.2) |  |
| **Suicidal attempt** |  |  | 0.852 |  |  | 0.700 |
| **No** | 5,269 (98.5) | 9,316 (98.5) |  | 5,255 (98.5) | 5,250 (98.4) |  |
| **Yes** | 83 (1.6) | 143 (1.5) |  | 83 (1.6) | 88 (1.7) |  |
| **Endocrine disorder (including thyroid disease)** |  |  | 0.210 |  |  | 0.837 |
| **No** | 4,884 (91.3) | 8,688 (91.9) |  | 4,873 (91.3) | 4,867 (91.2) |  |
| **Yes** | 468 (8.7) | 771 (8.2) |  | 465 (8.7) | 471 (8.8) |  |
| **Taking synthroid** |  |  | 0.699 |  |  | 0.737 |
| **No** | 5,191 (97.0) | 9,185 (97.1) |  | 5,177 (97.0) | 5,171 (96.9) |  |
| **Yes** | 161 (3.0) | 274 (2.9) |  | 161 (3.0) | 167 (3.1) |  |
| **Taking steroids** |  |  | 0.739 |  |  | 0.678 |
| **No** | 1,725 (32.2) | 3,074 (32.5) |  | 1,723 (32.3) | 1,703 (31.9) |  |
| **Yes** | 3,627 (67.8) | 6,385 (67.5) |  | 3,615 (67.7) | 3,635 (68.1) |  |
| **Diabetes** |  |  | 0.215 |  |  | 0.352 |
| **No** | 4,701 (87.8) | 8,373 (88.5) |  | 4,694 (87.9) | 4,725 (88.5) |  |
| **Yes** | 651 (12.2) | 1,086 (11.5) |  | 644 (12.1) | 613 (11.5) |  |
| **Hypertension** |  |  | 0.665 |  |  | 0.569 |
| **No** | 4,095 (76.5) | 7,267 (76.8) |  | 4,089 (76.6) | 4,064 (76.1) |  |
| **Yes** | 1,257 (23.5) | 2,192 (23.2) |  | 1,249 (23.4) | 1,274 (23.9) |  |
| **Hyperlipidemia** |  |  | 0.507 |  |  | 0.4036 |
| **No** | 4,381 (81.9) | 7,784 (82.3) |  | 4,372 (81.9) | 4,405 (82.5) |  |
| **Yes** | 971 (18.1) | 1,675 (17.7) |  | 966 (18.1) | 933 (17.5) |  |
| **COPD** |  |  | 0.851 |  |  | 0.187 |
| **No** | 5,160 (96.4) | 9,114 (96.4) |  | 5,149 (96.5) | 5,123 (96.0) |  |
| **Yes** | 192 (3.6) | 345 (3.7) |  | 189 (3.5) | 215 (4.0) |  |
| **CKD** |  |  | 0.112 |  |  | 0.365 |
| **No** | 5,324 (99.5) | 9,426 (99.7) |  | 5,313 (99.5) | 5,319 (99.6) |  |
| **Yes** | 28 (0.5) | 33 (0.4) |  | 25 (0.5) | 19 (0.4) |  |
| **LC** |  |  | 0.819 |  |  | >0.999 |
| **No** | 5,334 (99.7) | 9,425 (99.6) |  | 5,322 (99.7) | 5,322 (99.7) |  |
| **Yes** | 18 (0.3) | 34 (0.4) |  | 16 (0.3) | 16 (0.3) |  |
| **Heart failure** |  |  | 0.033 |  |  | 0.511 |
| **No** | 5,304 (99.1) | 9,403 (99.4) |  | 5,299 (99.3) | 5,293 (99.2) |  |
| **Yes** | 48 (0.9) | 56 (0.6) |  | 39 (0.7) | 45 (0.8) |  |
| **Chemotherapy** |  |  | 0.259 |  |  | 0.774 |
| **Not done** | 1,800 (33.6) | 3,268 (34.6) |  | 1,793 (33.6) | 1,779 (33.3) |  |
| **Done** | 3,552 (66.4) | 6,191 (65.5) |  | 3,545 (66.4) | 3,559 (66.7) |  |
| **Age (year, mean±SD)** | 51.07±11.5 | 50.73±10.47 | 0.071 | 51.04±11.48 | 51.14±10.98 | 0.640 |

COPD, chronic obstruction pulmonary disease; CKD, chronic kidney disease; LC, liver cirrhosis; SD, standard deviation

**Supplementary Table 11. Comparison of clinical characteristics of patients with invasive breast cancer according to taking tamoxifen**

|  | **Before matching** |  |  | **After matching** |  |  |
| --- | --- | --- | --- | --- | --- | --- |
|  | **Patients not receiving tamoxifen, n=5,352 (%)** | **Patients receiving tamoxifen, n=6,067 (%)** | ***P* value** | **Patients not receiving tamoxifen, n=3,877 (%)** | **Patients receiving tamoxifen, n=3,877 (%)** | ***P* value** |
| **Depression (only diagnosis)** |  |  | 0.076 |  |  | 0.470 |
| **No** | 4,482 (83.7) | 5,154 (85.0) |  | 3,288 (84.8) | 3,265 (84.2) |  |
| **Yes** | 870 (16.3) | 913 (15.1) |  | 589 (15.2) | 612 (15.8) |  |
| **Depression (diagnosis + anti-depressant)** |  |  | 0.353 |  |  | 0.245 |
| **No** | 4,654 (87.0) | 5,311 (87.5) |  | 3,405 (87.8) | 3,371 (87.0) |  |
| **Yes** | 698 (13.0) | 756 (12.5) |  | 472 (12.2) | 506 (13.1) |  |
| **Depression (only diagnosis) + suicidal attempt** |  |  | 0.353 |  |  | 0.512 |
| **No** | 5,339 (99.8) | 6,057 (99.8) |  | 3,865 (99.7) | 3,868 (99.8) |  |
| **Yes** | 13 (0.2) | 10 (0.2) |  | 12 (0.3) | 9 (0.2) |  |
| **Depression (diagnosis + anti-depressant) + suicidal attempt** |  |  | 0.167 |  |  | 0.251 |
| **No** | 5,339 (99.8) | 6,059 (99.9) |  | 3,865 (99.7) | 3,870 (99.8) |  |
| **Yes** | 13 (0.2) | 8 (0.1) |  | 12 (0.3) | 7 (0.2) |  |
| **Suicidal attempt** |  |  | 0.881 |  |  | 0.281 |
| **No** | 5,269 (98.5) | 5,975 (98.5) |  | 3,820 (98.5) | 3,808 (98.2) |  |
| **Yes** | 83 (1.6) | 92 (1.5) |  | 57 (1.5) | 69 (1.8) |  |
| **Endocrine disorder (including thyroid disease)** |  |  | 0.053 |  |  | 0.186 |
| **No** | 4,884 (91.3) | 5,597 (92.3) |  | 3,595 (92.7) | 3,564 (91.9) |  |
| **Yes** | 468 (8.7) | 470 (7.8) |  | 282 (7.3) | 313 (8.1) |  |
| **Taking synthroid** |  |  | 0.211 |  |  | 0.514 |
| **No** | 5,191 (97.0) | 5,908 (97.4) |  | 3,784 (97.6) | 3,775 (97.4) |  |
| **Yes** | 161 (3.0) | 159 (2.6) |  | 93 (2.4) | 102 (2.6) |  |
| **Taking steroids** |  |  | 0.049 |  |  | 0.790 |
| **No** | 1,725 (32.2) | 2,061 (34.0) |  | 1,269 (32.7) | 1,258 (32.5) |  |
| **Yes** | 3,627 (67.8) | 4,006 (66.0) |  | 2,608 (67.3) | 2,619 (67.6) |  |
| **Diabetes** |  |  | <0.001 |  |  | 0.968 |
| **No** | 4,701 (87.8) | 5,627 (92.8) |  | 3,542 (91.4) | 3,541 (91.3) |  |
| **Yes** | 651 (12.2) | 440 (7.3) |  | 335 (8.6) | 336 (8.7) |  |
| **Hypertension** |  |  | <0.001 |  |  | 0.226 |
| **No** | 4,095 (76.5) | 5,254 (86.6) |  | 3,279 (84.6) | 3,240 (83.6) |  |
| **Yes** | 1,257 (23.5) | 813 (13.4) |  | 598 (15.4) | 637 (16.4) |  |
| **Hyperlipidemia** |  |  | <0.001 |  |  | 0.645 |
| **No** | 4,381 (81.9) | 5,377 (88.6) |  | 3,349 (86.4) | 3,335 (86.0) |  |
| **Yes** | 971 (18.1) | 690 (11.4) |  | 528 (13.6) | 542 (14.0) |  |
| **COPD** |  |  | <0.001 |  |  | 0.784 |
| **No** | 5,160 (96.4) | 5,925 (97.7) |  | 3,766 (97.1) | 3,770 (97.2) |  |
| **Yes** | 192 (3.6) | 142 (2.3) |  | 111 (2.9) | 107 (2.8) |  |
| **CKD** |  |  | 0.039 |  |  | 0.563 |
| **No** | 5,324 (99.5) | 6,050 (99.7) |  | 3,862 (99.6) | 3,865 (99.7) |  |
| **Yes** | 28 (0.5) | 17 (0.3) |  | 15 (0.4) | 12 (0.3) |  |
| **LC** |  |  | 0.211 |  |  | >0.999 |
| **No** | 5,334 (99.7) | 6,054 (99.8) |  | 3,869 (99.8) | 3,869 (99.8) |  |
| **Yes** | 18 (0.3) | 13 (0.2) |  | 8 (0.2) | 8 (0.2) |  |
| **Heart failure** |  |  | <0.001 |  |  | 0.325 |
| **No** | 5,304 (99.1) | 6,044 (99.6) |  | 3,848 (99.3) | 3,855 (99.4) |  |
| **Yes** | 48 (0.9) | 23 (0.4) |  | 29 (0.8) | 22 (0.6) |  |
| **Chemotherapy** |  |  | 0.439 |  |  | 0.924 |
| **Not done** | 1,800 (33.6) | 1,999 (33.0) |  | 1,345 (34.7) | 1,341 (34.6) |  |
| **Done** | 3,552 (66.4) | 4,068 (67.1) |  | 2,532 (65.3) | 2,536 (65.4) |  |
| **Age (year, mean±SD)** | 51.07±11.5 | 45.96±8.43 | <0.001 | 47.26±9.97 | 47.22±9.87 | 0.863 |

COPD, chronic obstruction pulmonary disease; CKD, chronic kidney disease; LC, liver cirrhosis; SD, standard deviation

**Supplementary Table 12. Comparison of clinical characteristics of patients with invasive breast cancer according to taking aromatase inhibitor**

|  | **Before matching** |  |  | **After matching** |  |  |
| --- | --- | --- | --- | --- | --- | --- |
|  | **Patients not receiving aromatase inhibitor, n=5,352 (%)** | **Patients receiving aromatase inhibitor, n=3,310 (%)** | ***P* value** | **Patients not receiving aromatase inhibitor, n=2,780 (%)** | **Patients receiving aromatase inhibitor, n= 2,780(%)** | ***P* value** |
| **Depression (only diagnosis)** |  |  | 0.058 |  |  | 0.314 |
| **No** | 4,482 (83.7) | 2,720 (82.2) |  | 2,258 (81.2) | 2,287 (82.3) |  |
| **Yes** | 870 (16.3) | 590 (17.8) |  | 522 (18.8) | 493 (17.7) |  |
| **Depression (diagnosis + anti-depressant)** |  |  | 0.195 |  |  | 0.254 |
| **No** | 4,654 (87.0) | 2,846 (86.0) |  | 2,361 (84.9) | 2,391 (86.0) |  |
| **Yes** | 698 (13.0) | 464 (14.0) |  | 419 (15.1) | 389 (14.0) |  |
| **Depression (only diagnosis) + suicidal attempt** |  |  | 0.109 |  |  | 0.058 |
| **No** | 5,339 (99.8) | 3,307 (99.9) |  | 2,772 (99.7) | 2,778 (99.9) |  |
| **Yes** | 13 (0.2) | 3 (0.1) |  | 8 (0.3) | 2 (0.1) |  |
| **Depression (diagnosis + anti-depressant) + suicidal attempt** |  |  | 0.109 |  |  | 0.058 |
| **No** | 5,339 (99.8) | 3,307 (99.9) |  | 2,772 (99.7) | 2,778 (99.9) |  |
| **Yes** | 13 (0.2) | 3 (0.1) |  | 8 (0.3) | 2 (0.1) |  |
| **Suicidal attempt** |  |  | 0.626 |  |  | >0.999 |
| **No** | 5,269 (98.5) | 3,263 (98.6) |  | 2,741 (98.6) | 2,741 (98.6) |  |
| **Yes** | 83 (1.6) | 47 (1.4) |  | 39 (1.4) | 39 (1.4) |  |
| **Endocrine disorder (including thyroid disease)** |  |  | 0.789 |  |  | 0.778 |
| **No** | 4,884 (91.3) | 3,015 (91.1) |  | 2,528 (90.9) | 2,534 (91.2) |  |
| **Yes** | 468 (8.7) | 295 (8.9) |  | 252 (9.1) | 246 (8.9) |  |
| **Taking synthroid** |  |  | 0.331 |  |  | 0.714 |
| **No** | 5,191 (97.0) | 3,198 (96.6) |  | 2,681 (96.4) | 2,686 (96.6) |  |
| **Yes** | 161 (3.0) | 112 (3.4) |  | 99 (3.6) | 94 (3.4) |  |
| **Taking steroids** |  |  | 0.016 |  |  | 0.974 |
| **No** | 1,725 (32.2) | 985 (29.8) |  | 829 (29.8) | 828 (29.8) |  |
| **Yes** | 3,627 (67.8) | 2,325 (70.2) |  | 1,951 (70.2) | 1,952 (70.2) |  |
| **Diabetes** |  |  | <0.001 |  |  | 0.890 |
| **No** | 4,701 (87.8) | 2,688 (81.2) |  | 2,274 (81.8) | 2,270 (81.7) |  |
| **Yes** | 651 (12.2) | 622 (18.8) |  | 506 (18.2) | 510 (18.4) |  |
| **Hypertension** |  |  | <0.001 |  |  | 0.526 |
| **No** | 4,095 (76.5) | 1,965 (59.4) |  | 1,730 (62.2) | 1,707 (61.4) |  |
| **Yes** | 1,257 (23.5) | 1,345 (40.6) |  | 1,050 (37.8) | 1,073 (38.6) |  |
| **Hyperlipidemia** |  |  | <0.001 |  |  | 0.904 |
| **No** | 4,381 (81.9) | 2,347 (70.9) |  | 2,016 (72.5) | 2,020 (72.7) |  |
| **Yes** | 971 (18.1) | 963 (29.1) |  | 764 (27.5) | 760 (27.3) |  |
| **COPD** |  |  | <0.001 |  |  | 0.284 |
| **No** | 5,160 (96.4) | 3,111 (94.0) |  | 2,640 (95.0) | 2,622 (94.3) |  |
| **Yes** | 192 (3.6) | 199 (6.0) |  | 140 (5.0) | 158 (5.7) |  |
| **CKD** |  |  | 0.652 |  |  | 0.302 |
| **No** | 5,324 (99.5) | 3,295 (99.6) |  | 2,760 (99.3) | 2,766 (99.5) |  |
| **Yes** | 28 (0.5) | 15 (0.5) |  | 20 (0.7) | 14 (0.5) |  |
| **LC** |  |  | 0.067 |  |  | 0.723 |
| **No** | 5,334 (99.7) | 3,290 (99.4) |  | 2,765 (99.5) | 2,763 (99.4) |  |
| **Yes** | 18 (0.3) | 20 (0.6) |  | 15 (0.5) | 17 (0.6) |  |
| **Heart failure** |  |  | 0.850 |  |  | 0.222 |
| **No** | 5,304 (99.1) | 3,279 (99.1) |  | 2,741 (98.6) | 2,751 (99.0) |  |
| **Yes** | 48 (0.9) | 31 (0.9) |  | 39 (1.4) | 29 (1.0) |  |
| **Chemotherapy** |  |  | 0.001 |  |  | 0.451 |
| **Not done** | 1,800 (33.6) | 1,226 (37.0) |  | 991 (35.7) | 1,018 (36.6) |  |
| **Done** | 3,552 (66.4) | 2,084 (63.0) |  | 1,789 (64.4) | 1,762 (63.4) |  |
| **Age (year, mean±SD)** | 51.07±11.5 | 59.28±7.97 | <0.001 | 58.76±8.27 | 58.76±8.22 | 0.991 |

COPD, chronic obstruction pulmonary disease; CKD, chronic kidney disease; LC, liver cirrhosis; SD, standard deviation
